# Development of a Novel Homogeneous Liposome-Based One-Step Assay for SARS-CoV‑2 Antibody Detection in Human Serum Based on Fluorescent Liposomes and Complement Activity

**DOI:** 10.1021/acs.analchem.5c04506

**Published:** 2025-10-30

**Authors:** Christina Reiner, Kilian Hoecherl, Sebastian Einhauser, Simon Streif, Clemens Spitzenberg, Johannes Konrad, Patrick Neckermann, Miriam Breunig, Diana Pauly, Ralf Wagner, Antje J. Baeumner

**Affiliations:** † Institute of Analytical Chemistry, Chemo- and Biosensors, University of Regensburg, Universitaetsstr. 31, Regensburg 93053, Germany; ‡ Institute of Medical Microbiology & Hygiene, Molecular Microbiology (Virology), 210421 University of Regensburg, Franz-Joseph-Strauss-Allee 11, Regensburg 93053, Germany; § Department of Pharmaceutical Technology, 9147 University of Regensburg, Universitaetsstr. 31, Regensburg 93053, Germany; ∥ Experimental Ophthalmology, University of Marburg, Baldingerstr., Marburg 35043, Germany

## Abstract

Monitoring antibodies in patient serum enables the diagnosis of infectious and chronic diseases, the assessment of an individual’s immune status, and possible protection against infection and could thus be a ubiquitous tool for pandemic preparedness and personalized medicine. Advancing from the traditional enzyme-linked immunosorbent assay (ELISA), a homogeneous assay format was developed that simplifies assay procedures and enables a wash-free one-step performance. SARS-CoV-2 was chosen as the model virus, and its Spike protein-derived receptor binding domain (RBD) was covalently coupled to fluorescent liposomes. The binding of patient antibodies triggered the complement system, led to liposome lysis, and allowed quantitative fluorescent detection. The liposome assay was optimized with respect to liposome lipid composition, RBD coverage and surface chemistry, incubation conditions, and pretreatment of a standardized complement source. A proof-of-principle was demonstrated through artificially supplemented anti-RBD antibodies and full titrations with known positive sera. Testing of 37 SARS-CoV-2 negative sera and 28 sera from individuals with SARS-CoV-2 (breakthrough) infections resulted in a specificity of 95%, sensitivity of 93% and an excellent correlation (*R* ^2^ = 0.82, Spearman *r* = 0.90) with antibody titers determined in an ELISA approved for diagnostic use. Finally, the liposome assay showed a good correlation to a pseudovirus neutralization test (pVNT) (*R* ^2^ = 0.72, Spearman *r* = 0.84), similar to the diagnostic ELISA. As the new liposome assay does not require any wash steps and can be easily adapted to other viral targets by changing the surface antigen, it provides a new avenue for high-throughput immunodiagnostics.

## Graphical abstract

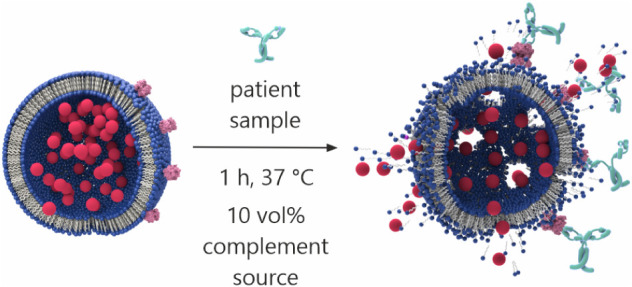

## Introduction

Antibody titers serve as correlates of protection (CoP) for many infectious diseases and allow statements about the success of a vaccination, an individual’s protection against the respective infection, and the herd immunity of a whole population.^1,2^ Furthermore, antibodies are not only used for infection monitoring^3–5^ but also as markers for the diagnosis of hereditary diseases, as they often arise in the context of autoimmune diseases^6^ like systemic lupus erythematosus,^7^ multiple sclerosis,^8^ Type I diabetes mellitus,^9^ or myasthenia gravis.^10^ In general, binding and neutralizing antibodies (nAbs) are distinguished with respect to their capability to directly neutralize, i.e., clear and interfere viral effects, for example, the binding to a cellular receptor that enables the uptake into the cell.^11^ nAbs are determined in virus-neutralization tests (VNTs), comprising plaque reduction neutralization tests (PRNTs), pseudovirus-based neutralization tests (pVNTs), and surrogate virus neutralization tests (sVNTs).^12^ PRNTs as the gold standard method for nAb detection provide excellent sensitivity and specificity but are time-consuming due to long incubation steps over several days, require biosafety level 3, and are not suited for a high-throughput format. pVNTs use nonreplicating pseudoviruses that exhibit an identical or highly relatable overall structure as the corresponding native virus and can therefore be conducted in biosafety level 2 laboratories, while providing highly comparable results to PRNTs.^13–15^ sVNTs without the need for any live viruses or cells are based on the nAb-mediated interference of the interaction of two proteins, one modified with an enzyme such as horseradish peroxidase (HRP) and can be used for large-scale testing due to their faster turnaround times.^16–18^ Several high-throughput assays as well as point-of-care (POC) formats have been developed for the detection of non-neutralizing IgG or IgM: ELISA approaches,^19^ Western blots,^20,21^ and immunofluorescence assays^22^ are used for the detection of ongoing or recent infections. These assays are often time-consuming and consist of several incubation and washing steps.

The severe acute respiratory syndrome coronavirus 2 (SARS-CoV-2) caused a global pandemic due to its high transmissibility and mutagenicity.^23,24^ After infection or vaccination, antibodies emerge as part of the humoral immune defense already after 7 days in case of IgM,^18,21,25^ followed by IgG, which are produced by antibody-secreting plasma cells after about 10–20 days.^22,25–27^ IgG can be used for the detection of a late stage of infection or postrecovery state, as they are still detectable after over 1 year.^18,21,28^ Many antibodies are targeted against the SARS-CoV-2 Spike protein containing the receptor binding domain (RBD), a sequence of 214 amino acids that plays a key role in the cell entry as it binds to the angiotensin-converting enzyme 2 (ACE2) as receptor.^24,29–32^ Most nAbs interfere with the ACE2 binding due to steric hindrance.^24,33^ The nAb titers allow statements on an individual’s immunity after infection or vaccination and a possible protection against a (re)­infection.^31,34,35^ Next to high-throughput ELISA formats^31,36,37^ and VNTs for the detection of nAbs,^13,14,17^ also POC formats have been established, mostly lateral flow assays (LFAs) using the Spike protein,^38^ RBD,^39,40^ or ACE2 ^41–44^ at the test line, resulting in an increase or decrease in signal in the presence of binding antibodies or nAbs, respectively. Similar assays were developed for influenza virus, with hemagglutination inhibition assays used for the detection of nAbs^45,46^ and ELISA-based assays employing the influenza membrane protein hemagglutinin as it contains the receptor binding site of the virus in its head domain.^45,47,48^ Antibodies against the respiratory syncytial virus (RSV) are detected in solid-phase immunoassays using the RSV fusion glycoprotein and cell-based serum neutralization assays.^49–51^ For the emerging Chikungunya virus (CHIKV) transmitted by mosquitoes and causing fever, headache, and muscle and joint pain, binding antibodies are determined in an ELISA using the envelope protein E2. High levels of binding and neutralizing antibodies against the envelope protein E2 are indicators of an ongoing or recent infection and correlate with long-term clinical protection.^52^ Consequently, there is a demand for highly sensitive and simple, wash-free assays with the potential for application at the POC for the detection of (neutralizing) antibodies in the context of diagnostics and disease prognosis. Here, a homogeneous liposome-based assay for the detection of anti-SARS-CoV-2 antibodies is introduced as a new platform technology that could be expanded to other viruses in the future.

Liposomes are spherical nanovesicles consisting of lipids arranged in a bilayer, enabling the encapsulation of an aqueous solution in their inner cavity and of hydrophobic molecules in the lipid membrane.^53,54^ The outer liposome surface can be modified with biological entities, e.g., nucleic acids or proteins to gain functionality.^44,55,56^ Therefore, liposomes are widely used for pharmaceutical applications (drug delivery,^57^ vaccination^58^ with much application as lipid nanocarriers for mRNA delivery during the COVID-19 pandemic^59^), industry (cosmetics,^60^ agriculture,^61^ food industry^62^), and biosensing.^53,63,64^ In the latter, liposomes are employed for signal amplification, resulting in better sensitivity due to the encapsulation of a multitude of label molecules^65,66^ or as simplified cell models.^67^ Various assay formats using liposomes have been developed, including heterogeneous assay formats for the investigation of liposome binding to an immobilized biorecognition element or homogeneous formats that do not require any washing steps.^53,68^

The new liposome-based assay for anti-SARS-CoV-2 antibody detection consists of no washing steps in contrast to laborious ELISA formats and provides quantitative results after only 1 h of incubation. Liposomes serve as mimics of biological membranes^52^ and are designed to quantitatively release a self-quenching fluorophore (sulforhodamine B) upon liposome lysis (Figure 1). Complement-induced liposome lysis is triggered by antibody binding to the antigen-decorated liposomal surface,^69^ here using the RBD of SARS-CoV-2 as the biorecognition element, which can be easily adapted to other viruses. The concept of complement-induced liposome lysis has been established and thoroughly described previously by Hoecherl et al.^70^ Thus, not only the binding but inherently also the complement-activating properties of the detected antibodies are determined. Endogenous complement activity of the patient sera is quenched by a simple heating step, and an external complement source with constant complement activity is applied. Finally, the new assay platform is compared to an ELISA and a pVNT assay and analyzed for its accuracy.

**Figure 1:**
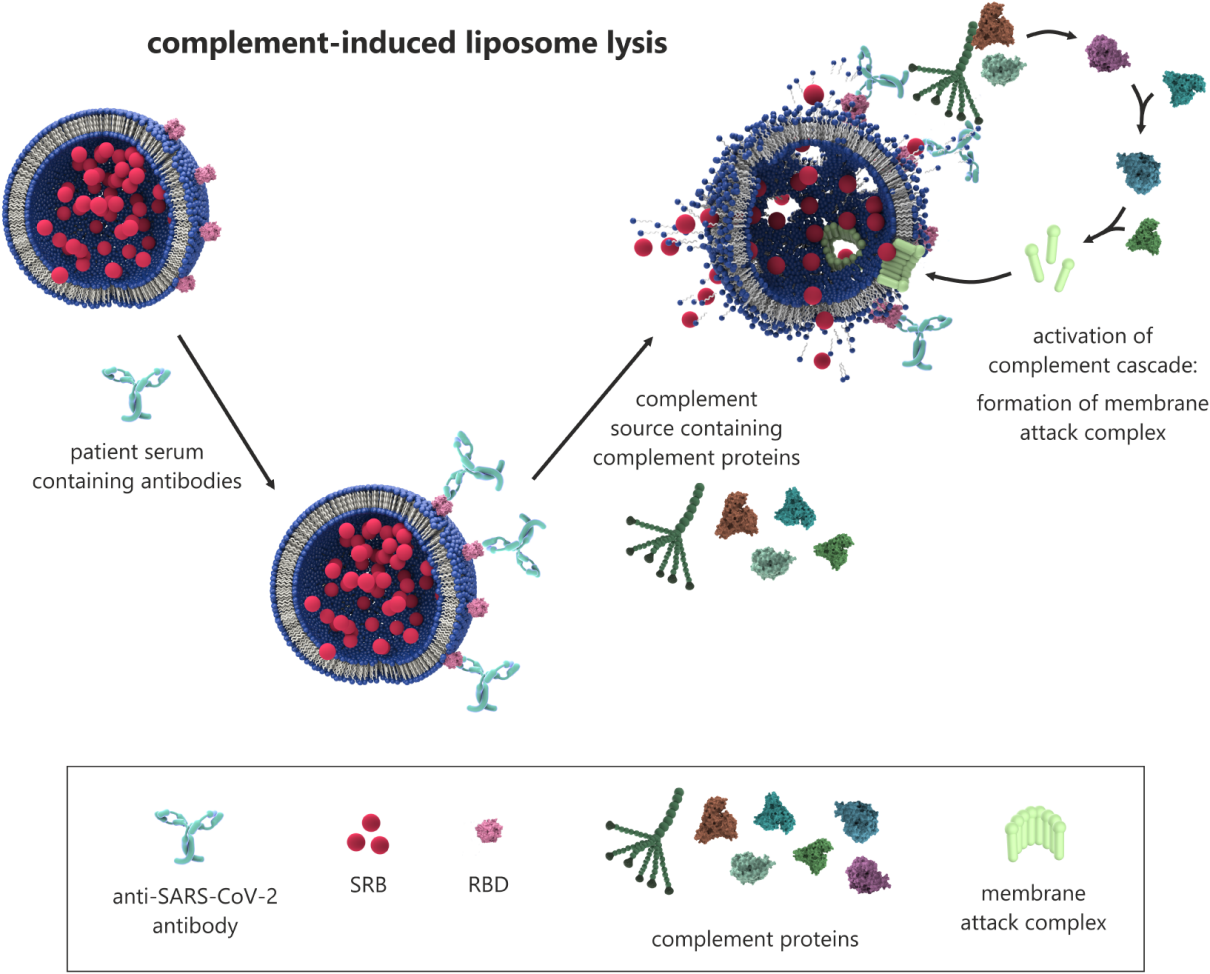
Schematic of the assay principle. Antibodies from a patient’s serum bind to liposomes, which are modified with the receptor binding domain (RBD) of SARS-CoV-2 and encapsulate the self-quenching fluorescent dye sulforhodamine B (SRB). As a result of this simple incubation step of liposomes with patient sera diluted in a liposome assay buffer and an external complement source, the complement system is activated, leading to the formation of the membrane attack complex and to pores in the lipid bilayer as well as the release of SRB, associated with an increase in the fluorescence intensity that is directly proportional to the antibody concentration.

## Experimental Section

### Chemicals and Consumables

All chemicals used were of analytical reagent grade. *n*-octyl-β-d-glucopyranoside (OG) (CN23, ≥98%), 4-(2-hydroxyethyl)-piperazine-1-ethanesulfonic acid (HEPES) (HN87, ≥99.5%), 2-(*N*-morpholino) ethanesulfonic acid (MES) (4259, ≥99%), d-(+)-sucrose, calcium chloride (CaCl_2_), potassium chloride (KCl), sodium chloride (NaCl), regenerated cellulose dialysis membrane Spectra/Por© 4 (MWCO: 12–14 kDa) (2718.1), and ROTIGarose Protein A Beads (1278, binding capacity for human IgG: 25 mg/mL) were purchased from Carl Roth (Karlsruhe, Germany); cholesterol from sheep wool (C8667, ≥99%), Sephadex G-50, sulforhodamine B (SRB) (230162), *N*-hydroxysulfosuccinimide sodium salt (sNHS) (≥98%), TWEEN 20, cysteamine dihydrochloride, tris­(hydroxymethyl)-aminomethane (TRIS), glycine, tetrasodium ethylenediaminetetraacetic acid (EDTA), ethylene glycol-bis­(β-aminoethyl ether)-*N*,*N*,*N*’,*N*’-tetraacetic acid (EGTA) from Sigma-Aldrich (Steinheim, Germany); 1,2-dipalmitoyl-*sn*-glycero-3-phosphoethanolamine-*N*-(glutaryl) (sodium salt) (*N*-glutaryl-DPPE) from Coatsome; 1,2-dipalmitoyl-*sn*-glycero-3-phosphocholine (DPPC), 1,2-dipalmitoyl-*sn*-glycero-3-phospho-(1′-*rac*-glycerol) (sodium salt) (DPPG), and the extruder set from Avanti Polar Lipids (Alabaster, USA); polycarbonate membranes (0.2, 0.4, and 1.0 μm pore size, Ø 19 mm) from Whatman (Dassel, Germany); sodium azide (NaN_3_), magnesium chloride hexahydrate (MgCl_2_), disodium hydrogen phosphate (Na_2_PO_4_), potassium dihydrogen phosphate (KH_2_PO_4_), sodium hydroxide (NaOH), hydrochloric acid (HCl), ethanol, *n*-butylamine, ethanolamine, and bovine serum albumin fraction V (BSA) from Merck (Darmstadt, Germany); l-lysine dihydrochloride from Fluka (Buchs, Switzerland); polyethylene glycol amine (PEGamine) from Jenkem Technologies (Spring Creek Pkwy, United States); polyclonal anti-RBD IgG antibody produced in rabbit (PA5–114451), 1-ethyl-3-(3-(dimethylamino)­propyl) carbodiimide hydrochloride (EDC) (PG82079), Nunc MaxiSorp black high-binding microplates (96-well, flat bottom), and black microtiter plates (96-well, flat, transparent bottom) from Thermo Scientific (Waltham, USA); black microtiter plates (96-well, flat, black bottom) from Brand (Wertheim, Germany); monoclonal human anti-RBD IgG1 antibody (S309) (BYT-ORB746635) from Biozol (Eching, Germany); chloroform, methanol, and Spectra-Por Float-A-Lyzer G2 (1 mL, MWCO: 1000 kDa) from Fisher Scientific (Schwerte, Germany); phosphorus standard for ICP measurements from Bernd Kraft (Duisburg, Germany); human complement sources (IRS31758, IRS38811, IRS41174, IRS45270, and IRS46827) and canine complement sources (#41378 and #43251) from Innovative Research (Novi, USA). SARS-CoV-2 RBD (Variant of Concern Alpha B1.1.7) and angiotensin-converting enzyme 2 (ACE2) were provided by Mikrogen (Neuried, Germany) and were produced according to the protocol described by Peterhoff et al.^31^ Monoclonal antibody CR3022 was expressed using the commercial Expi-Fectamine system (Thermo Fisher) according to manufacturer’s instructions and purified from cell culture supernatants using a HiTrap Protein A HP column (Cytiva) as described.^31^

SARS-CoV-2 positive sera (28 samples) were obtained using samples from the prospective longitudinal multicenter cohort study (CoVaKo) in which acute SARS-CoV-2 breakthrough infections (BTIs) and non-BTIs were analyzed. Study centers were the University Hospitals in Erlangen, Regensburg, Augsburg, Würzburg, and Munich (TUM and LMU), all located in Bavaria, Germany. The study design and cohort have been thoroughly described by Prelog et al.^71^ and Einhauser et al.^72^ To mimic the true population and avoid spectrum bias during test establishment,^37^ sera were selected for low to high spectrum of neutralizing antibody responses and variable immunization backgrounds, such as no, one, or two vaccinations, vaccination with Alpha or Delta breakthrough infection, or infection without previous vaccination. Seronegative samples were derived from the CoVaKo cohort (17 samples; early time points after infection) and from the Tirschenreuth-Kohorte-COVID-19 (TiKoCo) cohort^73,74^ (20 samples) and classified as negative based on the clinically approved ELISA^31^ result. The CoVaKo study was approved by the Ethics Committee of the Friedrich-Alexander-University Erlangen-Nürnberg, Germany (vote 46_21 B), and adopted by the local ethics committees of all other study centers. The CoVaKo Clinical Trials registration number was DRKS00024739. The TiKoCo study was approved by the Ethics Committee of the University of Regensburg, Germany (vote 20–1867–101), and adopted by the Ethics Committee of the University of Erlangen (vote 248_20 Bc). All study participants provided written informed consent. Both studies, TiKoCo and CoVaKo, comply with the 1964 Declaration of Helsinki and its later amendments.

### Liposome Synthesis

The reverse-phase evaporation method was used for liposome synthesis as described previously.^75^ For the encapsulant (4.5 mL), SRB and NaCl were dissolved in 20 mM HEPES, pH 7.5. Lipids were dissolved in 3 mL of chloroform and 0.5 mL of methanol, followed by sonication for 1 min. Two mL of the encapsulant were added to the lipid mixture and sonicated for 4 min at 60 °C. By stepwise reduction of pressure (900 mbar for 10 min, 850 mbar for 5 min, 800 mbar for 5 min, 780 mbar for 20 min), organic solvents were evaporated at 60 °C with a rotary evaporator (LABOROTA 4001 from Heidolph, Kelheim, Germany). The solution was vortexed for 1 min, 2 mL of encapsulant was added, and the solution was vortexed again for 1 min, followed by evaporation of the residual organic solvent at 60 °C (750 mbar for 20 min, 600 mbar for 5 min, 500 mbar for 5 min, 400 mbar for 20 min). The solution was extruded at 60 °C through polycarbonate membranes with pore sizes of 1, 0.4, and 0.2 μm by repeated pushing of the solution through syringes (21 repetitions each). Size exclusion chromatography with a Sephadex G-50 column was used for removal of the excess encapsulant, followed by dialysis overnight in a dialysis membrane Spectra/Por 4 (MWCO: 12–14 kDa) against HEPES-buffered saline with sucrose (HSS) consisting of 10 mM HEPES, 200 mM sucrose, 200 mM NaCl, and 0.01 w% sodium azide, pH 7.5.

### Liposome Characterization

The phospholipid concentration of the liposome solution was determined with inductively coupled plasma optical emission spectrometer (ICP-OES) measurements of the phosphorus concentration at λ = 177.495 nm (SpectroBlue TI/EOP from SPECTRO Analytical Instruments GmbH, Kleve, Germany). For the calibration of the device, phosphorus standard solution dilutions between 0 and 100 μM in 0.5 M HNO_3_ were used. Before each measurement, a recalibration was done with the 0 and 100 μM phosphorus dilutions. The liposome stock solution was diluted 1:150 in 0.5 M HNO_3_, and the total phosphorus content was determined. The total lipid content (tL) was calculated from the determined phosphorus concentration and the lipid composition used for the synthesis. The hydrodynamic diameter, polydispersity index (PDI), and zeta-potential of liposomes were determined by dynamic light scattering (DLS) measurements performed with a Malvern Zetasizer Nano-ZS (Malvern Panalytical, Malvern, United Kingdom). Liposomes were diluted to 25 μM tL in HSS (dispersant refractive index: n_D_^20^ = 1.34; dielectric constant: ε = 78.5; viscosity: η = 1.1185 cP). Poly­(methyl methacrylate) (PMMA) semimicro cuvettes (Brand, Wertheim, Germany) were used for size determination with an angle of 173° and backscattering mode after equilibration for 15 s at 25 °C in three measurement runs with each 13 single measurements. Disposable folded capillary cells (Malvern Panalytical, Malvern, United Kingdom) were used for zeta-potential measurements at 12.5 or 25 μM tL after equilibration at 25 °C for 60 s in four measurement runs with each 20 single measurements. The fluorescence of intact liposomes was determined in 1 μM tL of HSS, once with the addition of 30 mM detergent (OG) and once without, each in triplicates. The fluorescence was measured three consecutive times with a BioTek SYNERGY Neo2 fluorescence reader (Agilent Technologies, Santa Clara, USA) (λ_ex_ = 560 nm and λ_em_ = 585 nm, bandwidth (BW) = 10 nm, gain 100, measurement from the top). The so-called initial fluorescence was calculated as the ratio of the fluorescence intensities of intact and lysed liposomes.

### Surface Modification of Liposomes with RBD

RBD was conjugated to liposomes via EDC/sNHS chemistry by using carboxyl groups on the outer liposome surface. EDC and sNHS in 50 mM MES buffer were added to the desired amount of liposomes (1:100:180 ratio of carboxyl groups:EDC:sNHS) followed by incubation for 1 h at room temperature (RT) and 300 rpm. Afterward, the RBD was added, and the solution was incubated for 1.5 h at RT and 300 rpm. For removal of the excess coupling reagents, dialysis was performed after the coupling. A Spectra/Por Float-A-Lyzer G2 (1 mL, MWCO: 1000 kDa) was prepared by rinsing three times with ethanol (10 vol %), double-distilled water, and HSS, each for 10 min. Afterward, the reaction mixture was added to the Spectra/Por Float-A-Lyzer G2 and dialyzed against HSS for 20–23 h with three buffer exchanges. The total lipid concentration was determined by ICP-OES measurement, and the protein-modified liposomes were stored at 4 °C.

### Homogeneous Liposome Assay

Four conditions were investigated in triplicates: liposomes in (reactivation) liposome complement buffer ((R)­LCB) only, liposomes with active complement source (aS), liposomes with complement source that was inactivated (iaS) by the addition of an inactivation complement buffer (iaCB, LCB containing 200 mM EDTA and 0.5 mM EGTA) as a negative control, and liposomes with complement source and the detergent OG (30 mM) for complete liposome lysis as a positive control. Patient sera were handled in a biosafety cabinet in a biosafety level II laboratory, and the patient complement system was initially heat-inactivated at 56 °C for 30 min and 300 rpm. Liposomes were diluted to 10 μM tL in HSS and incubated with patient serum at 37 °C for 20 min. In the case of anti-RBD antibody used instead of human serum, liposomes were incubated with 0.2 or 0.5 mol % antibody (RT, 60 min, 300 rpm). LCB contained 10 mM HEPES, 150 mM NaCl, 135 nM CaCl_2_, and 1 mM MgCl_2_ at pH 7.4. RLCB was prepared from LCB by the addition of 1.07 mM CaCl_2_ and 2.15 mM MgCl_2_. LCB or RLCB (30–70 μL per well), sucrose in LCB (20 μL per well), iaCB (10 μL in respective wells), and 300 mM OG (10 μL in respective wells) were added to a black microtiter plate (MTP) with a flat, black, or transparent bottom. Liposome solution was added (10 μL per well), resulting in 1 μM tL. Complement source was added to the aS, iaS, and OG samples, resulting in 10 vol % complement source and a total volume of 100 μL per well. In each assay, an internal control was included using the same human serum. The MTP was sealed with transparent adhesive foil before removal from the biosafety cabinet to prevent aerosol formation. Fluorescence was measured three consecutive times in 1.5 min intervals for the first 15 min followed by 5 min intervals for another 45 min. Fluorescence measurements were performed three consecutive times with a BioTek SYNERGY Neo2 fluorescence reader (λ_ex_ = 565 nm, λ_em_ = 585 nm, BW = 8 nm, gain 150, measurement from the top or from the bottom). Alternatively, liposomes were lysed after the measurement to save on patient samples by the addition of OG (300 mM, 10 μL per well) and incubation for 15 min at RT and 300 rpm. The fluorescence was measured three consecutive times with the same settings.

### Heterogeneous Binding Assay

ACE2 (5 μg/mL in phosphate-buffered saline (PBS) consisting of 10 mM Na_2_HPO_4_, 1.8 mM KH_2_PO_4_, 137 mM NaCl, and 2.7 mM KCl, pH 7.4; 100 μL) was immobilized in a Nunc MaxiSorp high-binding microplate (Thermo Fisher) overnight at 4 °C. The solution was removed, and the plate was blocked with BSA (1 w/v% in PBS-T, 150 μL) for 1 h at RT and 300 rpm. The plate was washed two times with PBS-T (consisting of PBS with 0.1 w % TWEEN 20) and three times with HSS (each 150 μL) before the addition of RBD-liposomes (10 μM tL in HSS, 100 μL) and incubation for 3 h at RT and 300 rpm. The plate was washed three times with HSS (150 μL), and bound liposomes were lysed by 15 min incubation at RT and 300 rpm with 30 mM OG in double-distilled water (100 μL). The fluorescence was measured three consecutive times with a BioTek SYNERGY Neo2 fluorescence reader (λ_ex_ = 560 nm and λ_em_ = 585 nm, BW = 10 nm, gain = 150, measurement from the top).

### Preparation of Human Complement Source

A commercially available human complement source was purified from interfering antibodies by incubation with agarose beads bearing protein A on their surface. Before the purification procedure, the complement source was temporarily inactivated by the addition of EDTA (16 mM per 100 vol %) to prevent complement activation during the procedure and therefore a loss in complement activity. Protein A beads were washed with Tris buffer (50 mM, pH 7.0) and centrifuged at 3350 × *g* for 5 min. These steps were repeated three times. All of the following centrifugation steps were performed with the same settings. The inactivated complement source was incubated with protein A beads (ratio of approximately 1:0.8 of the initial volume of protein A beads (50% suspension) to complement source) for 2 h at 4 °C and 500 rpm. The suspension was centrifuged, and the supernatant, i.e., the antibody-purified complement source, was collected and stored at −80 °C. Protein A beads were regenerated by washing with Tris buffer (3x), 10 min incubation at RT with glycine (100 mM, pH 3.0) for elution of antibodies (2x), washing with double-distilled water (3x) and with 20 vol % ethanol (2x). The regenerated beads were stored in 20 vol % ethanol at 4 °C. The dilution of the purified complement source in Tris buffer was quantified by absorbance measurement compared to a calibration curve with known serum dilutions with a BioTek SYNERGY Neo2 absorbance reader (λ = 450 nm, three consecutive measurements).

### ELISA

Two slightly different protocols were utilized for the ELISA. First, for SARS-CoV-2 RBD wildtype (D614G), the clinically validated in-house method was performed as described previously.^31^ In brief, Nunc Maxisorp plates (Thermo Fisher) were coated with SARS-CoV-2 RBD protein (WT), blocked with fat-free milk, and primarily stained using triplicates of a 1:100 dilution of human sera in 1% milk for 1 h. After incubation with the secondary antibody and subsequent detection with TMB, plates were read for OD 450 nm on a microplate reader. Finally, signal-to-cutoff values were used as the readout. This assay was referred to as “diagnostic ELISA”.

Second, for RBD Alpha, the protocol was adjusted to use a 5-fold dilution series of sera starting at 1:20 instead of a single-point measurement. EC_50_ values were then determined using a four-parameter logistic regression (Algorithm “[agonist] vs. response”) in GraphPad Prism 8.0.1. The protocol was adjusted for Alpha to provide a more robust quantitative readout than single-point measurements and account for variant-specific potential changes in the correlations and cutoffs determined by Peterhoff et al.^31^ This assay was referred to as “variant matched fully titrated ELISA”.

### Pseudovirus Neutralization Test (pVNT)

The pseudovirus neutralization for SARS-CoV-2 was performed as described previously.^72,76^ In brief, an inoculum containing 2.5 × 10^5^ RLU after 48 h/384-well of lentiviral particles expressing luciferase and pseudotyped with the SARS-CoV-2 Spike protein was neutralized using a 2-fold serum dilution series starting at 1:20 for 1 h. After 48 h of infection of HEK293T-ACE2+ cells, luciferase activity was determined using Bright-Glo reagent (Promega Corp., Madison, WI, USA). The 50% inhibitory dilution (ID_50_) of the sera was calculated in GraphPad Prism 8.0.1 by normalizing the data to both infected and noninfected cells, followed by curve fitting with the “log (inhibitor) vs normalized response” algorithm. Neutralizing antibody titers were assessed against various SARS-CoV-2 variants, namely WT (D614G), Alpha (B.1.1.7), Delta (B.1.617.2), and Omicron BA1, BA2, and BA5. ID_50_ values of 2561 denote 100% assay saturation at the lowest tested dilution of 2560.

### Testing of Anti-Influenza Cross-Reactivity

The Influenza (IgG)-ELISA was adapted from the detailed protocol described in Peterhoff et al.^31^ In brief, 350 ng/well Influenza HA protein (A/England/195/2009) in PBS was coated on Nunc Maxisorp plates overnight, blocked 1 h with 5% skim milk powder, primarily stained with a 4-fold serum dilution starting at 1:50, and secondarily stained with polyclonal rabbit anti-Human IgG-HRP (Agilent Technologies, P0214) in a 1:5000 dilution. OD at 450 nm was determined after 2 min of TMB incubation and stopping with 1 M H_2_SO_4_. Titration curves for each serum were determined in three independent measurements and normalized to the mean PBS signal and the highest signal of the positive (recently Influenza-infected) control serum on each plate.

### Data Evaluation

All data are presented as the mean ± standard deviation (SD). The raw data were processed as follows: The fluorescence intensities (*I*) at 60 min of the aS condition and the positive control were corrected for the background by subtracting the *I* of the negative control (iaS). The ratio of these corrected intensities called “lysis signal” was calculated as $\mathrm{lysis signal}=\frac{I\left(aS,60\min\right)-I\left(iaS,60\min\right)}{I\left(\mathrm{positive control},60\min\right)-I\left(iaS,60\min\right)}\times 100\%$ . EC_50_ values and 95% confidence intervals (CIs) were determined from dose–response curves (lysis signal dependent on vol % serum) fitted with the “logistic fit” function including four nonfixed parameters (initial and final value, center, power = 3) with Levenberg–Marquardt iteration algorithm and instrumental weighting performed with Origin 2022b, version 9.9.5.167, OriginLab Corporation (Northampton, USA). Finally, EC_50_ values were transformed to serum dilution factors ED_50_ as ${\mathrm{ED}}_{50}=\frac{100}{{\mathrm{EC}}_{50}\left[\mathrm{vol}\%\right]}$ . Samples were determined as seronegative when the lysis signal of the highest sample concentration was below the cutoff. It was determined as the mean lysis signal of all negative sample concentrations plus three times the standard deviation, resulting in 8% lysis signal as the cutoff value. The coefficient of variation (CV) between all assays was calculated as the ratio of the standard deviation and arithmetic mean of the lysis signals of the internal control.

Statistical analyses were performed using GraphPad Prism 8.0.1 (GraphPad Software, Boston, Massachusetts, USA). Residuals of linear correlated ELISA, pVNT, and liposome assay data were tested for Gaussian distribution (Shapiro–Wilk test, *p* ≥ 0.05) and for homoscedasticity (*p* ≥ 0.05) as requirements for a linear fit. Data were logarithmically transformed with a base of 2 or 5, as a 2- or 5-fold dilution series was performed in the pVNT or ELISA, respectively, to fulfill the conditions for linear fitting (ELISA and liposome-based assay: Shapiro–Wilk: *p* = 0.083; homoscedasticity: *p* = 0.4101; pVNT and liposome-based assay: Shapiro–Wilk: *p* = 0.4992; homoscedasticity: *p* = 0.2910). Spearman *r* of the correlations between ELISA or pVNT and liposome-based assay was calculated with two-tailed α = 0.05 (both *p* < 0.0001). Liposome storage conditions were compared using two-tailed, paired, or unpaired Student’s *t* tests (confidence level: 95%). The comparisons of different liposome compositions and modifications were performed by two-way analyses of variance (ANOVAs) with posthoc Tukey’s or Dunnett’s test, α = 0.05. Statistical significance was displayed as *p*-values, where ns, *, **, ***, and **** correspond to nonsignificant, *p* < 0.05, *p* < 0.01, *p* < 0.001, and *p* < 0.0001, respectively.

Receiver operating characteristic (ROC) analysis was performed to evaluate the binary discriminative ability of the liposome-based assay against the binary outcome, as determined by the diagnostic ELISA. For dichotomous classification, the maximum mean liposome lysis signal for any sample concentration was used. The area under the ROC curve (AUC) was calculated as a measure of the overall classification performance, and the optimal cutoff was determined by Youden’s J (sensitivity + specificity – 1) on a fine threshold grid (1000 thresholds over the score range), selecting the cutoff to maximize the statistic. Confidence intervals and uncertainty for the ROC, AUC, optimal cutoff, and connected sensitivity and specificity were estimated by 2000 bootstrap resamples. Analysis and plotting were performed in Python (3.12.11) using Pandas (2.3.1), NumPy (2.0.1), scikit-learn (1.7.1), and Matplotlib (3.10.0).

## Results and Discussion

A novel homogeneous liposome-based assay for the quantification of anti-SARS-CoV-2 antibodies in serum samples was developed, which does not require immobilization or washing steps and provides results after only 1 h. It is based on liposomes covalently tagged with RBD and entrapping the fluorescent, self-quenching dye SRB. When anti-SARS-CoV-2 antibodies bind to the liposomes, lysis occurs due to complement activation, leading to the release of SRB and a quantitative increase of the fluorescence intensity. In the absence of a complement trigger, liposomes remain intact due to their stealth behavior, which means they are stable in the presence of a complement source. The liposomes were optimized regarding surface modification chemistry, coverage with RBD, and their long-term stability. Further assay development was performed in terms of incubation conditions, amount of complement source, and treatment strategies for commercially available complement sources containing anti-SARS-CoV-2 antibodies to avoid false-positive signals. For the proof-of-principle, several commercially available anti-RBD antibodies targeting different epitopes and seropositive human serum were used. To demonstrate actual applicability, a serum panel consisting of 28 seropositive and 37 seronegative patient samples was screened to determine assay sensitivity and specificity as well as correlation with an ELISA and a pVNT. The serum panel was specifically chosen to mimic the variety of the population’s immunization background and avoid potential bias during test establishment.^37^ Hence, positive sera were selected for a wide range of neutralizing antibody responses, different time points postinfection, and a multitude of different immunization backgrounds, e.g., (breakthrough) infection with different variants (Delta, Alpha) and different vaccination backgrounds (0x, 1x, 2x). Negative sera for cutoff determination were selected from two different cohorts established during the early pandemic and selected based on a negative result in a diagnostically validated in-house ELISA.^31^

### Optimization of Long-Term Stable RBD-Liposomes

Liposomes were synthesized following an established protocol and modified with RBD using EDC/sNHS chemistry.^75^ To identify the optimal surface coverage, concentrations between 0.1 and 1 mol % of RBD per total lipid concentration were coupled. The resulting liposomes were characterized physically using DLS to determine the increase in the hydrodynamic diameter and zeta-potential with increasing amounts of conjugated protein. A plateau starting at 0.4 mol % RBD was observed, indicating maximum occupancy of the liposome surface (Figure S1). The biological functionality was investigated by quantifying the specific binding of liposomes to immobilized ACE2 in a microtiter plate (Figure 2A) as well as their lysability by the complement system in the presence of 0.2 mol % anti-RBD antibody (Figure 2B). Not surprisingly, at the lower concentration range (<0.4 mol %), it was found that the more RBD was coupled to the liposomes, the better binding to ACE2 was observed. At concentrations >0.6 mol %, less binding was seen, which is assumed to be caused by cross-linking of the protein and therefore a decrease in accessible RBD binding sites on the liposomal surface. Liposome modification with 0.5 mol % RBD was found to be optimal, resulting in a maximum in complement-induced lysis and a plateau in binding to ACE2. It should be noted that the RBD concentration described refers to the initially calculated concentration. It was not feasible with the equipment available to determine the actual protein amount per liposome. This shortcoming could be addressed in the future via radionuclide-labeled RBD to determine the coupling efficiency.

**Figure 2:**
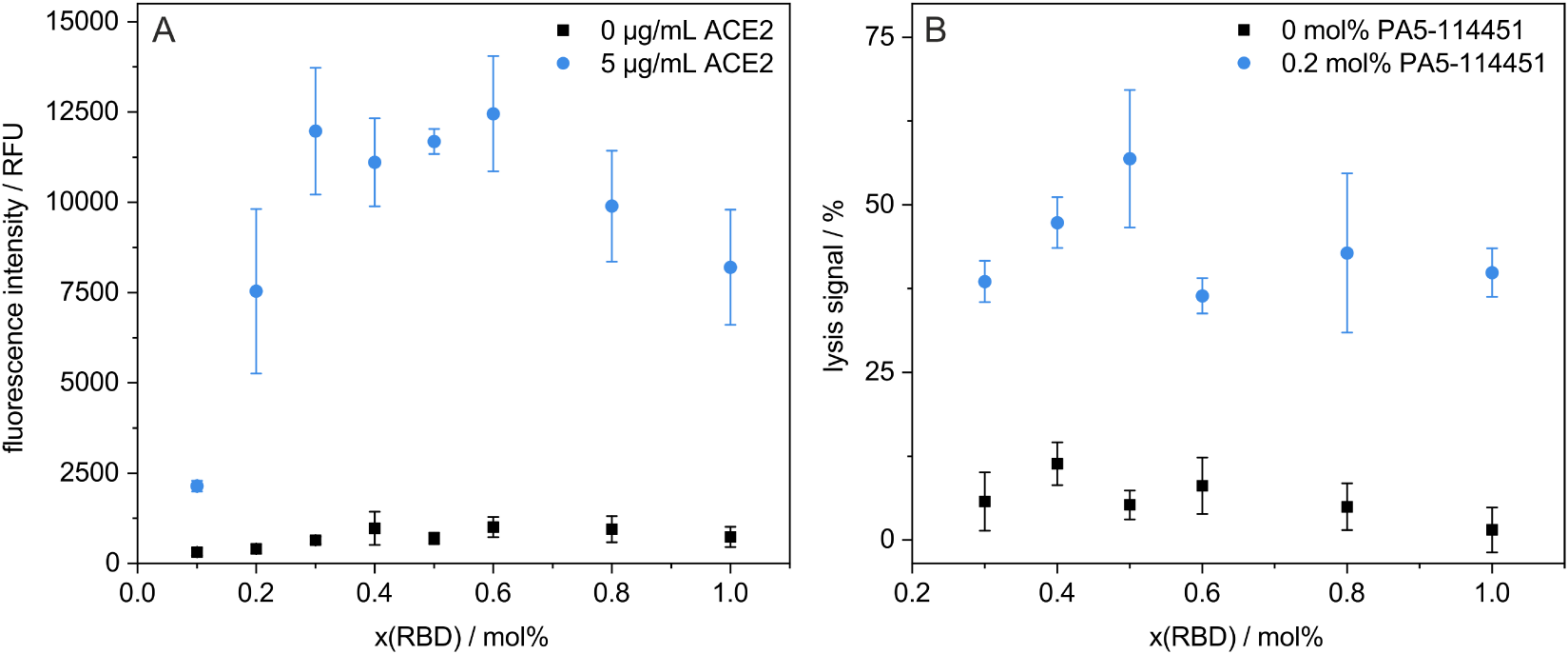
Effects of different RBD concentrations on the liposome surface were studied: (A) Binding of RBD-liposomes to immobilized 0 or 5 μg/mL ACE2, incubated for 3 h at RT and 300 rpm. *n* = 3. Nonbound liposomes were washed away. Bound liposomes were lysed by a detergent, with their fluorescence signal recorded. (B) Complement-induced lysis with 0 or 0.2 mol % neutralizing anti-RBD antibody PA5-114451. Liposomes were incubated for 60 min at 37 °C in the presence of 10 vol % of a canine complement source. The lysis signals were calculated as described in the Data Evaluation section. Shown are the mean ± SD, *n* = 3.

With respect to the lipid composition, earlier studies had identified the influence of cholesterol on complement-induced liposome lysis.^70^ Thus, a range between 5 and 20 mol % cholesterol was investigated (Figure S2) in combination with RBD-surface coverage examining its potential enhancing or shielding effect. It was demonstrated that liposomes with 20 mol % cholesterol and more did significantly lose stability in the complement source irrespective of protein coverage (*p* = 0.011 for liposomes with 42 mol % cholesterol). Surprisingly, liposomes were significantly more prone to complement-induced lysis after EDC/sNHS-based coupling even at lower cholesterol concentrations (*p* = 0.012). To confirm that the remaining reactivity on the liposome surface could play a role in this phenomenon, a range of quenching molecules was studied to saturate the excess surface carboxyl groups remaining activated after the RBD conjugation step (Figure S3). Whereas PEGamine, ethanolamine, *n*-butylamine, l-lysine, and glycine were found to have no statistically significant effect (0.24 < *p* < 0.99), cysteamine resulted in significantly more nonspecific complement-induced lysis independent of the cholesterol content of the liposomes (5 mol %: *p* = 0.0004, 10 mol %: *p* < 0.0001). On that basis, it was decided to quench through natural hydrolysis of the activated groups avoiding unpredictable responses and simplifying the overall process.

These optimized liposomes modified with RBD were found to be long-term stable against agglomeration and leakage of the encapsulant for at least 1 year when stored with 0.05 wt % BSA at 4 °C, as no significant increase was observed for size (*p* = 0.17), PDI (*p* = 0.11) (Figure S4A), and initial fluorescence (*p* = 0.050) as measures of SRB leakage and colloidal stability (Figure S4B). While bare or otherwise-modified liposomes are known to be long-term stable,^66,77^ it was found that in the absence of the stabilizing agent BSA, RBD-liposomes start to agglomerate quickly as indicated by a significant increase in size (*p* = 0.027 (4 weeks), *p* = 0.0030 (52 weeks)) and PDI (*p* = 0.0002 (4 weeks), *p* = 0.0005 (52 weeks)) already after 4 weeks. The presence of 0.01–0.05 wt % BSA had no significant effect on the liposome fluorescence properties at day zero (maximum fluorescence intensity: *p* = 0.56, initial fluorescence: *p* = 0.85) (Figure S4B), the complement-induced lysis at all time points (*p* = 0.37) (Figure S5A) nor their binding to ACE2 (*p* = 0.67) (Figure S5B). Whereas it is known that proteins that have undergone coupling chemistries^78–80^ often show tendencies of denaturing or agglomeration, our findings suggest that BSA addition enhances the colloidal stability of RBD-modified liposomes, similar to previous findings with HSA.^81^ Further studies on the effect of storage temperature, freezing, lyophilization, and transport stress would be required for the commercial application of the assay.

### Proof of Principle: Complement-Induced Liposome Lysis by Antibody Binding to Liposomes

The general assay principle was demonstrated using the neutralizing anti-RBD antibodies (nAb) polyclonal IgG PA5–114451 and monoclonal IgG1 antibody S309, monoclonal binding IgG1 antibody CR3022, and neutralizing human serum (Figure 3A). IgG1 antibodies were chosen as model antibodies since they are known to be efficient complement activators, while IgG2 is only weakly activating and IgG4 is often considered to be incapable of activating the complement system.^82,83^ Results were as expected and corresponded to the published binding and neutralization activities of the antibodies: The polyclonal nAb PA5-114451 resulted in strong positive signals, whereas the monoclonal nAb S309 (*K*_d_ = 0.555 nM)^84^ showed only a low effect. The non-neutralizing CR3022 (*K*_d_ = 0.27 nM)^31,85^ did not show any complement-induced lysis at all. Differences in complement activation between polyclonal and high-affinity monoclonal antibodies could be attributed to the higher density of polyclonal antibodies bound to the antigen compared with monoclonal ones, yielding avidity effects for complement activation. This also applies to the combination of S309 and CR3022 leading to strong positive signals and revealing synergistic effects on complement-induced lysis as previously observed for antibodies targeting different epitopes^70^ and also for the neutralizing activity of anti-SARS-CoV-2 antibodies in general.^86^ In this assay, not explicitly neutralizing antibodies are detected but rather binding antibodies with a complement activation potential. Whereas neutralization depends on the interference of antibodies with viral binding to the cellular receptor and/or the prevention of membrane penetration,^26^ the capability of classical pathway activation relies on the antibody subtype^87^ and local density.^88^ In fact, complement activation is often mediated by neutralizing antibodies,^87,89^ with some antibodies relying on complement activation for complete neutralization.^88^ However, also non-neutralizing antibodies have been shown to be able to trigger the complement cascade.^87,89^

**Figure 3:**
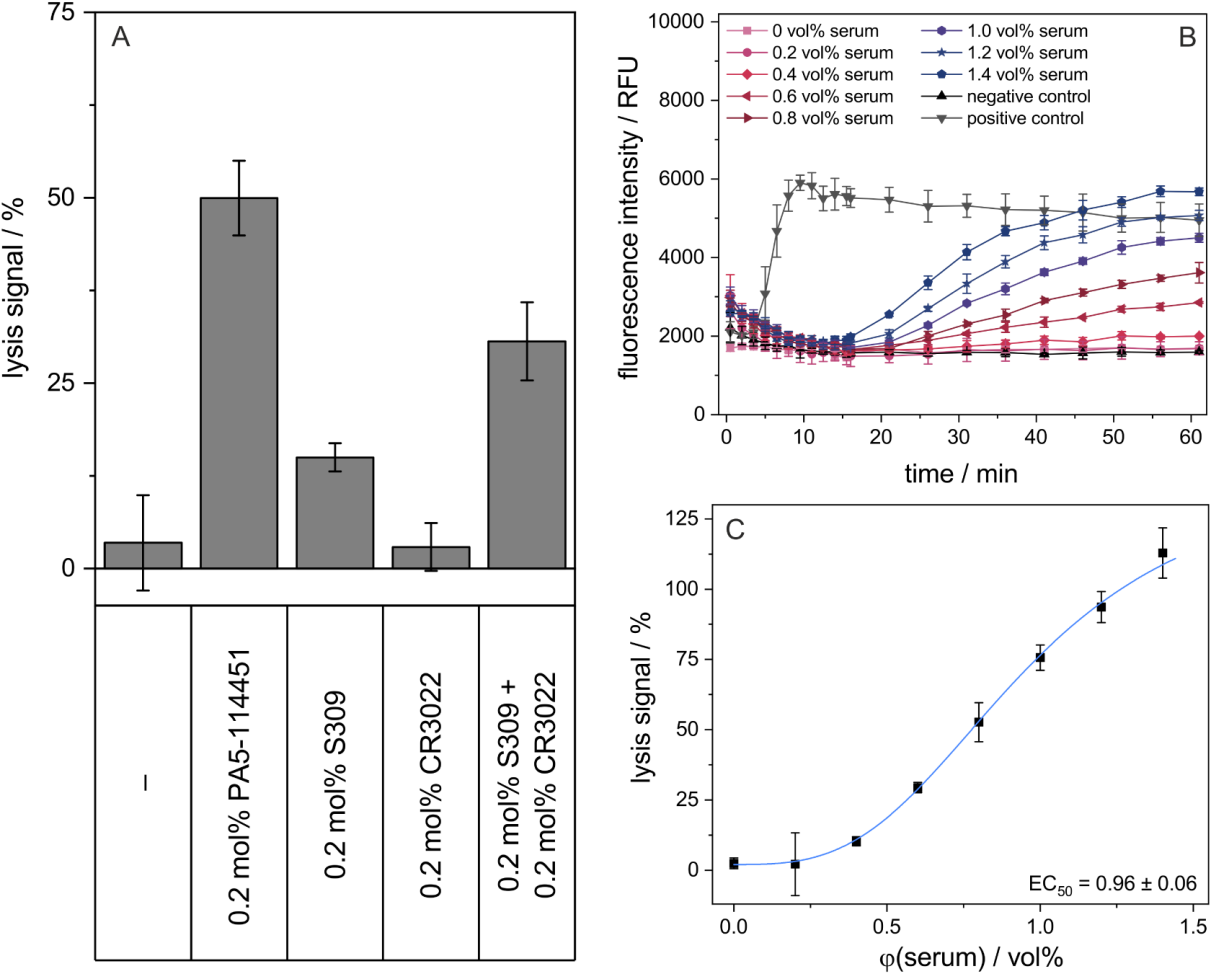
Proof-of-principle of the liposome-based assay was performed with commercially available antibodies and neutralizing human serum. (A) Complement-induced lysis of stealth liposomes by 0.2 mol % antibodies PA5-114451 (neutralizing), S309 (neutralizing), and CR3022 (non-neutralizing) and a combination of S309 and CR3022, each at 0.2 mol %, demonstrating synergistic effects for the combination of antibodies with various epitopes. RBD-liposomes were preincubated with the respective antibody for 1 h at RT and 300 rpm. Liposomes were then incubated for 60 min at 37 °C in the presence of 10 vol % of a canine complement source. The lysis signals were calculated as described in Data Evaluation section. Shown are mean ± SD, *n* = 3. (B) Varying the serum concentration between 0 and 1.4 vol %. The raw dataset is shown, resulting in a time-resolved graph demonstrating complement-induced lysis of the liposomes starting at 15 min. RBD-liposomes were preincubated with serum for 20 min at 37 °C. Liposomes were then incubated for 60 min at 37 °C in the presence of 10 vol % of a human complement source with 3 μg/mL RBD. Inactivated complement source was used as a negative control, and liposomes fully lysed by a detergent were used as a positive control. Shown are mean ± SD, *n* = 3. (C) Endpoint lysis signals at 60 min were calculated as described in Data Evaluation section for the time-resolved dataset in (B). EC_50_ values and 95% CIs were determined using a 4-parameter logistic regression curve fit as described in Data Evaluation section. Shown are mean ± SD, *n* = 3.

In the case of neutralizing human serum, increasing concentrations resulted in an increasing extent of complement-induced lysis, allowing the calculation of an EC_50_ value of such samples as a measure of the antibody amount in the serum (Figure 3B,C). In the absence of antibodies, liposomes did not show lysis and remained stealth (Figure 3A–C).

### Optimization of Liposome Assay Conditions

For the development of a rugged assay, patient sample handling, treatment of the complement source, and incubation conditions were studied and optimized.

Patient samples showed strongly varying complement activity, indicating the need for an external complement source to allow accurate quantification of antibody titers. In case of highly active patient samples, liposome lysis occurred during the incubation step leading to elevated background signals in the negative control with increasing sample concentration (Figures S6 and S7). Further, liposome lysis was caused even in the absence of the external complement source, confirming the interference of the sample (Figure S8). To exclude these variations in the total complement activity, which would deliver noncomparable results, the complement proteins in the patient sample were denatured through a simple heat inactivation at 56 °C for 30 min. This treatment has been shown to preserve antibody functionality, as demonstrated by Hu et al.^90^

Currently, commercially available human complement sources contain anti-SARS-CoV-2 antibodies; at present, most of the donors are either vaccinated or had an infection. Therefore, it cannot be used without further treatment for the detection of these antibodies, as its use would result in false-positive signals. A canine complement source was found to be suited as an interim alternative for the assay optimization, enabling complement-induced lysis in the presence of a neutralizing antibody in a broad concentration range (2–10 vol %) while maintaining liposome stealthiness in its absence (Figure S9A,B). Yet, to provide a sustainable alternative also for other virus detection assays in the future, independence of any antibody present in the complement source and avoidance of adverse species-related interactions are desirable. Thus, two additional strategies have been developed to enable the use of a human complement source even if it contains anti-SARS-CoV-2 (or other) antibodies.

In a first rapid and simple strategy, antibodies were captured by the addition of free RBD, hence successfully preventing their binding to RBD-liposomes. No enhancing or decreasing effects on complement-induced lysis were observed when compared to the use of a prepandemic human complement source (Figure 4A). Though there were no undesired competition effects using this protocol, it should be noted that they theoretically cannot be fully excluded in case of different affinities of antibodies in the complement source and antibodies in the patient sample. Hence, as a more rigid method, the human complement source was cleared from all antibodies through protein A-bearing agarose beads. The removal of anti-RBD antibodies was confirmed as no liposome lysis was observed in the absence of a serum sample (Figure 4B). Unfortunately, this more rigid method resulted in dramatically decreased complement activity (∼50%) which had to be compensated by an increased amount of the complement source per assay. To overcome this wasteful use of reagents and to protect the complement activity during the purification process, complement activation was prevented through the addition of EDTA chelating Ca^2+^ and Mg^2+^ ions (Figure S10). After the purification process, the complement source was reactivated by the quantitative addition of Ca^2+^ and Mg^2+^, resulting in the reconstitution of ∼90% of the initial complement activity (Figure 4B). Normalization of different batches of complement sources can easily be performed by comparison with the untreated source. Minimizing this need for normalization is guaranteed by processing larger batches at once.

**Figure 4:**
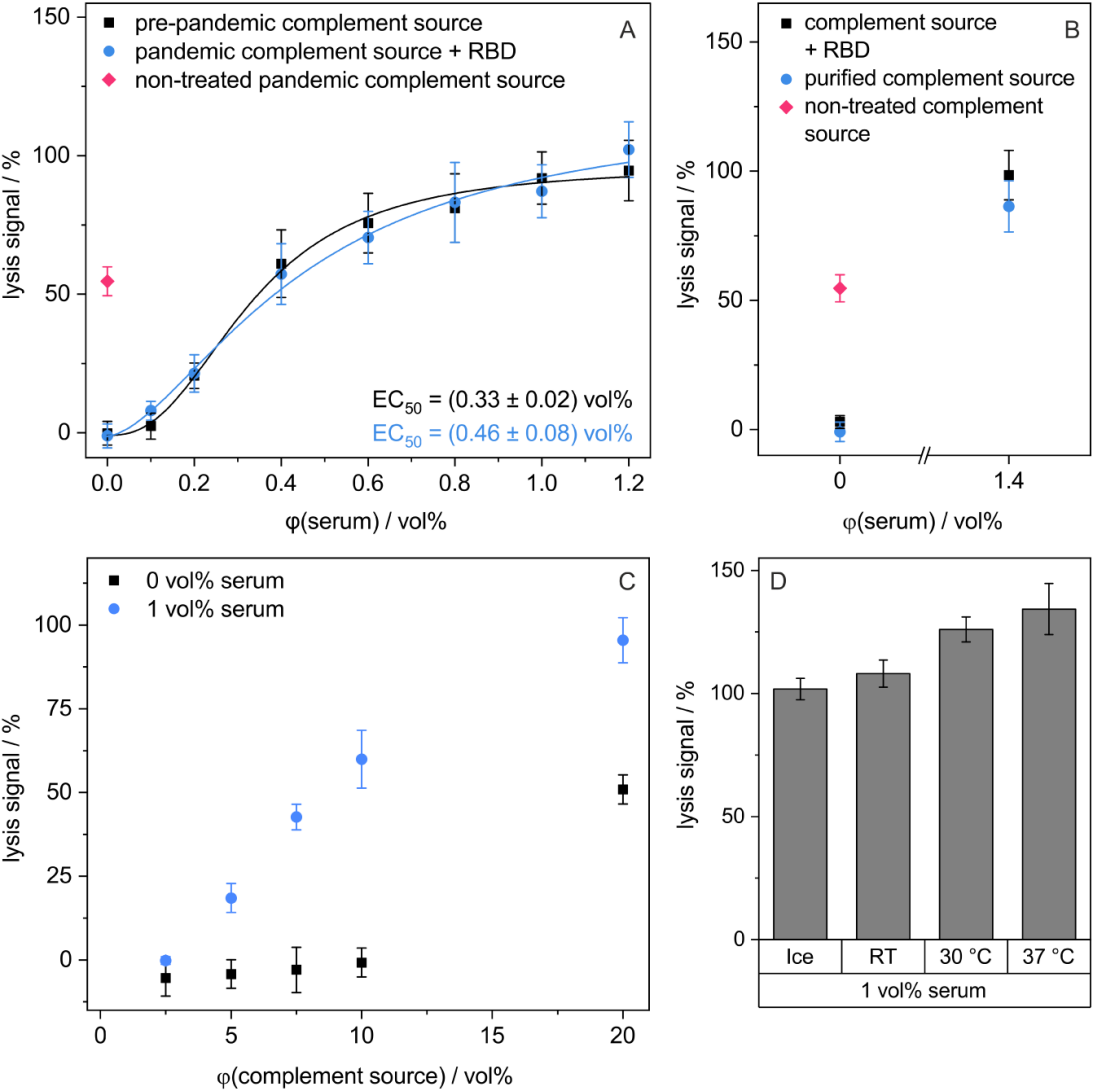
Assay was optimized regarding the complement source and incubation conditions. (A) Treatment of the pandemic complement source by incubation with 4.5 μg/mL free RBD for 60 min on ice to prevent the binding of antibodies to RBD-liposomes. RBD-liposomes were preincubated with serum for 20 min at 37 °C. Liposomes were then incubated for 60 min at 37 °C in the presence of 10 vol % of the respective complement source. EC_50_ values and 95% CIs were determined using a 4-parameter logistic regression curve fit as described in Data Evaluation section. (B) Purification of pandemic complement source from anti-SARS-CoV-2 antibodies with protein A, combined with interim inactivation (1.6 mM EDTA) and reactivation (1.1 mM Ca^2+^, 0.5 Mg^2+^). RBD-liposomes were preincubated with serum for 20 min at 37 °C. Liposomes were then incubated for 60 min at 37 °C in the presence of 10 vol % of the respective complement source. (C) Optimization of the concentration of human complement source treated with protein A between 2.5 and 20 vol % for maximum complement-induced lysis while maintaining the liposome stealthiness. RBD-liposomes were preincubated with serum for 20 min at 37 °C. Liposomes were then incubated for 60 min at 37 °C in the presence of 2.5–20 vol % of complement source treated with protein A. (D) Complement-induced lysis after 20 min of preincubation of RBD-liposomes with 1 vol % serum on ice, at RT, 30 or 37 °C. Liposomes were incubated for 60 min at 37 °C in the presence of 10 vol % of human complement source with 4.5 μg/mL RBD. (A-D) The lysis signals were calculated as described in Data Evaluation section. Shown are mean ± SD, *n* = 3.

Hence, protein A purification in combination with interim complement deactivation and activation was chosen as the favorable method, as it is independent of varying anti-SARS-CoV-2 antibody concentrations, more rigid toward unwanted competition effects, and applicable for other future bioassay strategies without being limited to SARS-CoV-2-based assays.

Next, the amount of complement source was optimized to maximize complement-induced lysis of liposomes in the presence of neutralizing serum while maintaining the stealthiness of the liposomes in the absence of patient serum to enhance assay sensitivity (Figure 4C). Increasing complement-induced lysis was observed with increasing complement source concentration up to 10 vol % as the best condition. For higher amounts of complement source, the liposomes were not stealthy and showed nonspecific lysis^70^ which also led to a decrease in the assay range (Figure S11). 37 °C was found to be the best incubation temperature for the 20 min incubation of liposomes with patient serum, as it led to more complement-induced lysis compared to the incubation on ice, at RT and at 30 °C (Figure 4D). This could be attributed to enhanced diffusion and interaction of antibodies and RBD-liposomes at higher temperatures. As previously observed, signals above 100% were due to a decrease in the positive control over time as exemplarily seen in Figure 3B, which is caused by detergent-derived quenching of the fluorophore.^70^ As no major changes in the extent of the decrease have been observed, it was decided to perform the normalization to the endpoint rather than manually selecting the respective maximum for normalization.

### Testing Patient Samples

In total, 37 seronegative samples were successfully screened and resulted in a specificity of 95% as two of the samples showed low false-positive lysis signals (13% and 16%) in the highest sample concentration tested (Figure S13). No interferences were observed for samples from patients with autoimmune or cardiovascular diseases, as well as cancer (Table S1). Also, no cross-reactivity of anti-Influenza A (A/England/195/2009 H1) was observed (Figure S12). In previous work,^31^ the cross-reactivity of seasonal CoV-positive sera was investigated in SARS-CoV-2-specific ELISAs, and no cross-reactive binding was detected, suggesting no cross-reactive complement activation. Twenty-eight seropositive samples resulted in dose–response curves from which an EC_50_ was determined for 26 samples (Figure S14). The remaining two samples showed false-negative results, leading to a sensitivity of 93%. ROC curve analysis (Figure S17) with bootstrapping showed excellent discriminative ability of the assay compared to the diagnostically used ELISA, with an AUC of 0.99 (95% CI: 0.98–1.00). The optimal cutoff value, determined using Youden’s J statistic, was 16.3% (95% CI: 3.74–16.33) lysis in the highest sample dilution, with corresponding sensitivity of 92.6% (95% CI: 88.0–100%) and specificity of 100% (95% CI: 87.5–100%). This could likely be further improved by the use of higher sample concentrations, liposome modification with the RBD of the current variant of concern, or the oriented immobilization of biotinylated RBD on streptavidin-/neutravidin-modified liposomes instead of a random coupling strategy, as previously shown by Streif et al.^81^ Specifically, site-directed modification would further reduce interbatch variabilities occurring due to the random conjugation of RBD to the liposomes. Furthermore, the liposome assay was benchmarked for its quantitative connection with established test formats, such as the clinically validated single-point measurement ELISA (as widely used in diagnostics), a variant-matched fully titrated ELISA (as often used in research), and a variant-matched pVNT. Excellent correlations with the diagnostic ELISA (*R* ^2^ = 0.80, Spearman *r* = 0.90 (Figure S15)) and with the variant-matched fully titrated ELISA (*R* ^2^ = 0.82, Spearman *r* = 0.90 (Figure 5A)) were observed. As expected, the correlation with pVNT data was lower (*R* ^2^ = 0.72, Spearman *r* = 0.84, Figure 5B) but still solid and was in the same range as the correlation between ELISA and pVNT.^31,91^ A coefficient of variation of 11% was determined from 22 assays using the same standard serum as a positive control, proving low interassay variation and high reproducibility of the assay (Figure S16).

**Figure 5:**
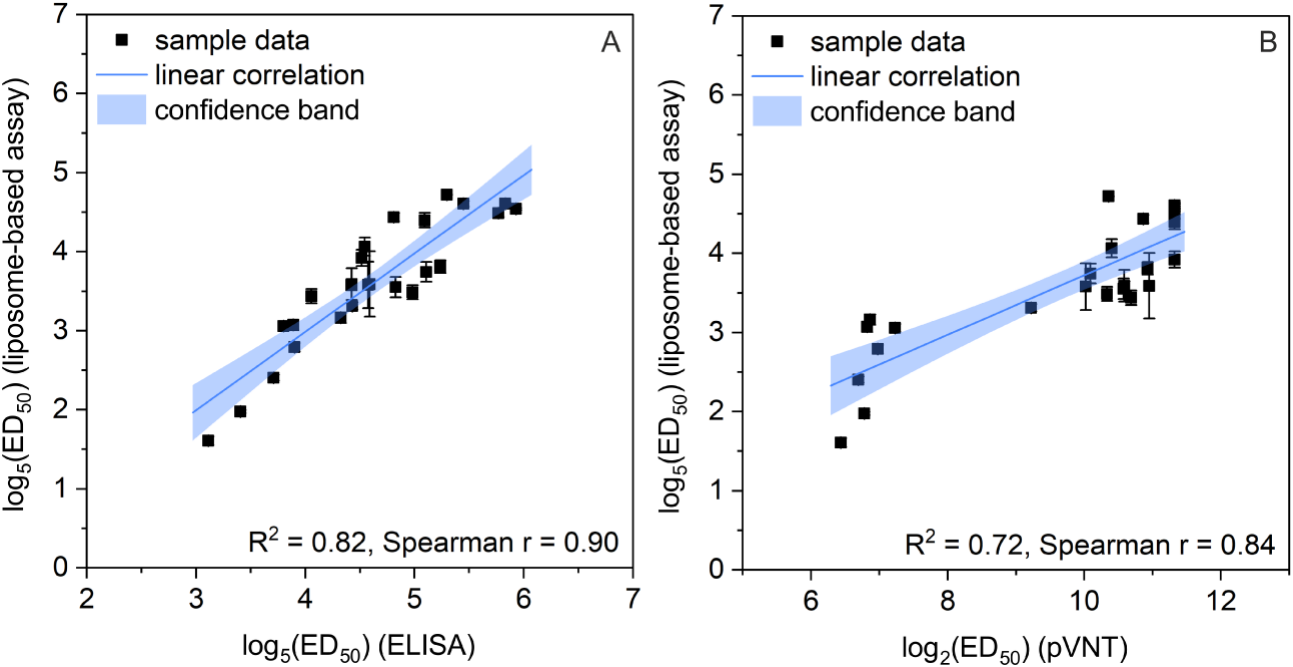
Twenty-eight seropositive human serum samples were screened and correlated to other assay formats. (A) Correlation of log_5_(ED_50_) values from the liposome-based assay with the log_5_(ED_50_) values from the variant-matched fully titrated ELISA, confidence level = 0.95. (B) Correlation of log_5_(ED_50_) values from the liposome-based assay with the log_2_(ED_50_) values from the pVNT, confidence level = 0.95.

## Conclusion

Antibody titers are often determined in time-consuming and laborious assay formats including multiple incubation and washing steps like in neutralization tests or ELISAs or give only qualitative results as in many LFAs. The liposome-based assay developed here provides a novel wash-free diagnostic platform for antibody detection and quantification, relying on the virus-mimicking properties of liposomes and their lysability in the presence of a complement source activated by an antibody bound to their phospholipid membrane. Therefore, not only can the binding properties of antibodies be monitored but inherently also their capability of activating the complement system, which has been shown to be of great importance in antiviral response, for example, in neutralization assays^88^ for the quantification of antibodies against SARS-CoV-2,^92^ measles,^93^ RSV,^94^ and HIV.^95^

The homogeneous, wash-free format presented here enables rapid signal development and minimizes handling steps, making the assay well-suited for adaptation to a lateral flow assay format by capturing intact liposomes on a test line and separating them from lysed ones.^70^ As the assay can be easily adapted for other viral targets in the future by changing the surface antigen, our liposome platform provides a new avenue for high-throughput, decentralized immunodiagnostics. Even multiplexed testing for various antibodies could be possible with different encapsulants and liposome surface modifications. The assay format could furthermore provide deeper insights into the interaction of protecting antibodies with the patient-specific complement system, if the inherent complement activity of the patient serum is used for liposome lysis instead of an external complement source, enabling a better understanding of the individual effects in viral protection as well as complement-associated autoimmune diseases such as systemic lupus erythematosus, membranous nephropathy, or myasthenia gravis.^96^

## Supplementary Material



## Notes

Additional information regarding liposome characteristics and sample sera, additional figures providing more details on the assay optimization performed (complement inactivation of patient samples by heat treatment, alternative complement sources, concentration of complement source), serological screening, and long-term storage of liposomes (PDF)
