# Supplementary material for: Development of a Novel Homogeneous Liposome-Based One-Step Assay for SARS-CoV‑2 Antibody Detection in Human Serum Based on Fluorescent Liposomes and Complement Activity
Source: Anal Chem. 2025 Oct 30;97(44):24543–55. doi: 10.1021/acs.analchem.5c04506 (PMC12613147; doi:10.1021/acs.analchem.5c04506)
Supplement: Supplementary file 1 [file ac5c04506_si_001.pdf]

Supporting Information

Development of a novel homogeneous liposome-based one-step assay for SARS-CoV-2 antibody detection in human serum based on fluorescent liposomes and complement activity

Christina Reiner[1a], Kilian Hoecherl[1a], Sebastian Einhauser[2a], Simon Streif[1], Clemens Spitzenberg[1], Johannes Konrad[3], Patrick Neckermann[2], Miriam Breunig[3], Diana Pauly[4], Ralf Wagner[2], Antje J. Baeumner*[1]

[1] Institute of Analytical Chemistry, Chemo- and Biosensors, University of Regensburg, Universitaetsstr. 31, 93053 Regensburg, Germany
[2] Institute of Medical Microbiology & Hygiene, Molecular Microbiology (Virology), University of Regensburg, Franz-Joseph-Strauss-Allee 11, 93053 Regensburg
[3] Department of Pharmaceutical Technology, University of Regensburg, Universitaetsstr. 31, 93053 Regensburg, Germany
[4] Experimental Ophthalmology, University of Marburg, Baldingerstr., 35043 Marburg

[a] equal contribution
* antje.baeumner@ur.de

Table of Contents

Size and Zeta-potential of RBD-liposomes 2
Optimization of Liposome Cholesterol Content 2
Quencher Study 3
Long-Term Storage of RBD-liposomes 3
Effect of BSA added to Liposomes on Complement-induced Lysis and Binding to ACE2 4
Patient Sample Complement Activity 4
Heat-Inactivation of the Patient Sample 6
Canine Complement Source as Potential Alternative for Pandemic Human Source 6
Inactivation and Reactivation of Human Complement Source 7
Assay Range Depending on the Concentration of Complement Source 7
Seronegative Samples 8
Seropositive Samples 9
Receiver Operating Characteristic (ROC) Curve 13

Size and Zeta-potential of RBD-liposomes

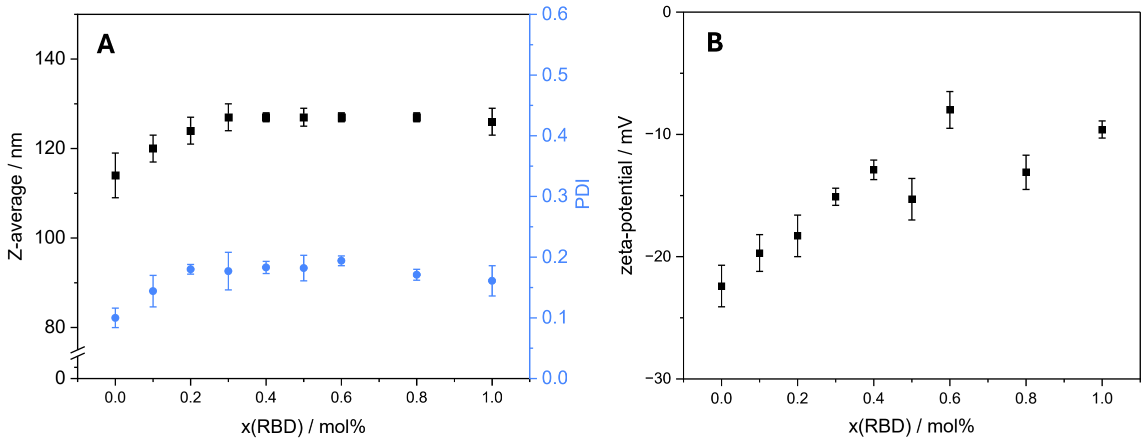

Figure S1: Physical properties of liposomes modified with 0, 0.1, 0.2, 0.3, 0.4, 0.5, 0.6, 0.8 and 1 mol% RBD via EDC/sNHS chemistry. (A) Z-average and polydispersity index PDI, mean and standard deviation determined from 3 individual runs à 13 measurements. (B) Zeta-potential, determined from 4 individual runs à 20 measurements. Shown are mean ± SD.

Optimization of Liposome Cholesterol Content

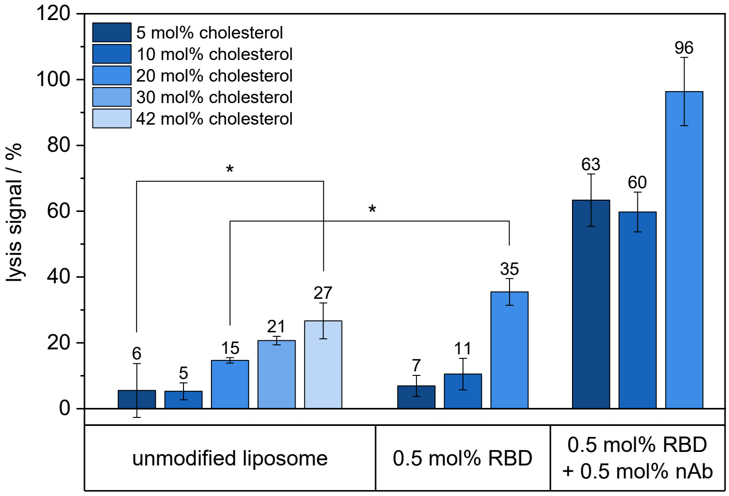

Figure S2: The cholesterol content of liposomes was investigated in a range between 5 and 42 mol% of the total lipid composition. Either the unmodified liposomes or liposomes modified with 0.5 mol% RBD were investigated. The latter were incubated with or without 0.5 mol% neutralizing anti-RBD antibody PA5-114451 (nAb) for 60 min at RT and 300 rpm. Liposomes were then incubated for 60 min at 37 °C in the presence of 10 vol% of canine complement source. The lysis signals were calculated as described in section Data Evaluation. Two-way ANOVA including post-hoc Tukey's test for significance determination. Shown are mean ± SD, n = 3.

Quencher Study

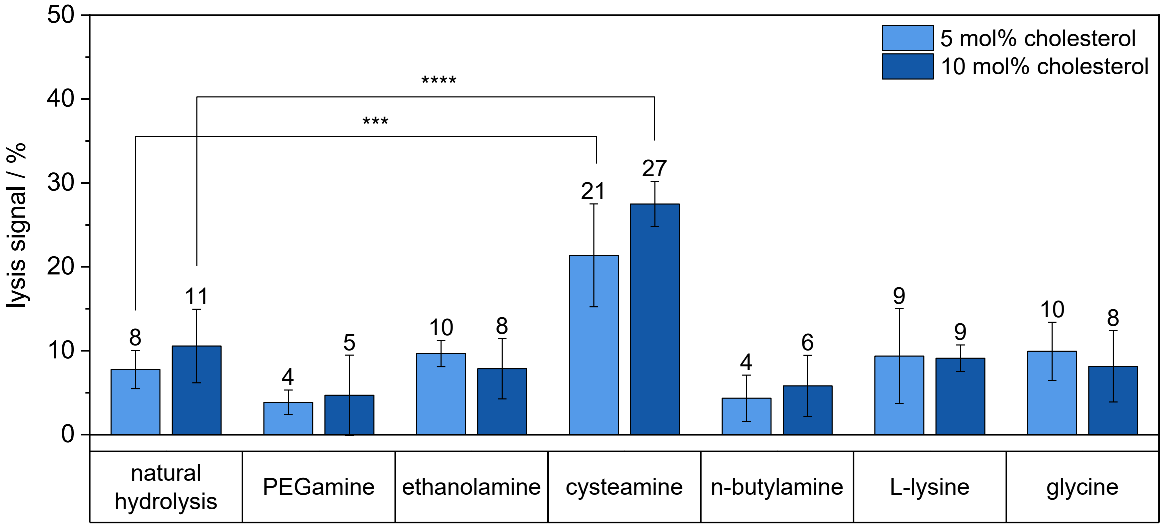

Figure S3: The use of amines as quenchers after the conjugation of protein to liposomes was investigated for PEGamine, ethanolamine, cysteamine, n-butylamine, L-lysine and glycine. For comparison, no quencher, i.e., spontaneous hydrolysis, and non-treated liposomes without surface activation were used. Liposomes were incubated for 60 min at 37 °C in the presence of 10 vol% of canine complement source. The lysis signals were calculated as described in section Data Evaluation. Two-way ANOVA including post-hoc Dunnett's test for significance determination. Shown are mean ± SD, n = 3.

Long-Term Storage of RBD-liposomes

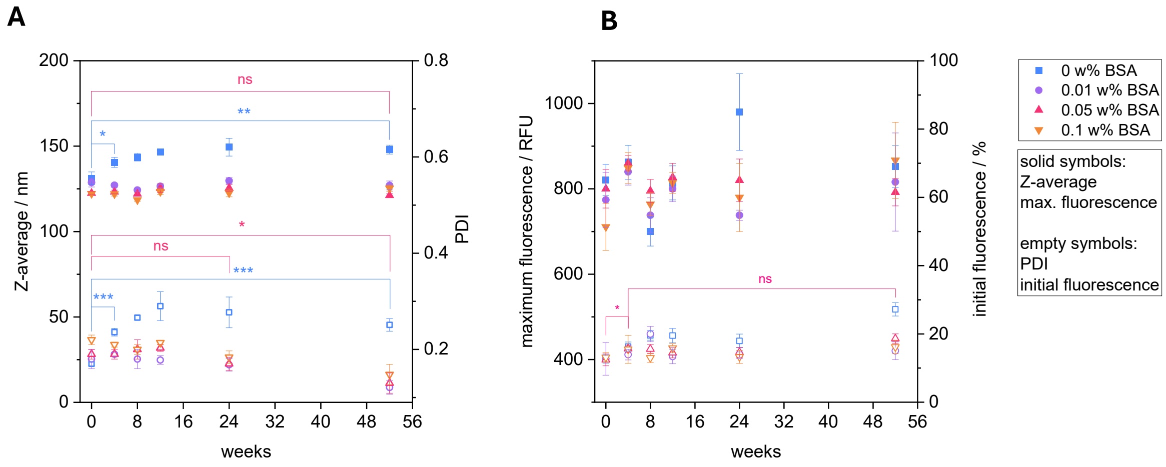

Figure S4: The long-term stability of RBD-liposomes stored with 0, 0.01, 0.05 and 0.1 w% BSA was investigated over one year. (A) Z-average and polydispersity index (PDI) indicate agglomeration of liposomes stored without BSA after 4 weeks. No significant increase in the PDI was observed within the first 24 weeks ($p = 0.11$). A significant decrease ($p = 0.011$) was observed after 52 weeks. As the PDIs were slightly lower at this time for all BSA conditions compared to the previous time points, this was attributed to a general variation in the measurement and not to a trend leading to liposomes with a higher monodispersity. (B) Maximum fluorescence intensity of liposomes fully lysed by a detergent and initial fluorescence of unlysed liposomes, normalized to the maximum fluorescence. No significant changes of maximum ($p = 0.56$) and initial fluorescence ($p = 0.84$) were observed between liposomes stored with 0.01-0.05 w% BSA or without at day zero. A significant increase ($p = 0.046$) in the initial fluorescence was observed within the first 4 weeks for liposomes stored with 0.05 w% BSA. No significant increase ($p = 0.050$) was observed between 4 and 52 weeks. Two-tailed, unpaired t-test for significance determination. Mean ± SD, n = 3.

Effect of BSA added to Liposomes on Complement-induced Lysis and Binding to ACE2

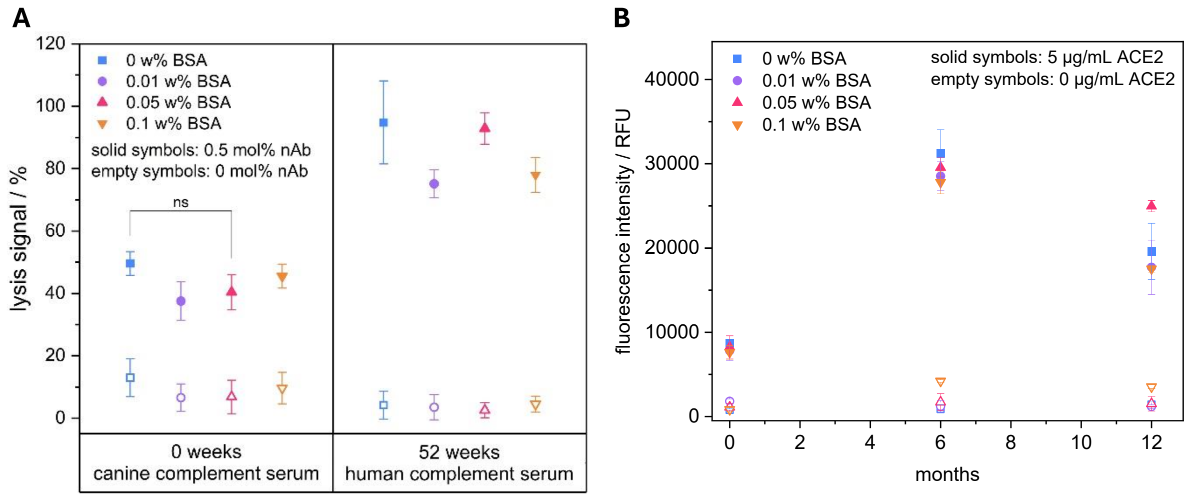

Figure S5: The effect of BSA (0 w%, 0.01 w%, 0.05 w% and 0.1 w%) added for better long-time stability of liposomes on the complement-induced liposome lysis and the binding to ACE2 was investigated over one year. (A) Complement-induced lysis with 0 or 0.5 mol% neutralizing anti-RBD antibody PA5-114451. Liposomes were incubated for 60 min at 37 °C in the presence of 10 vol% canine complement source (0 months) or human complement source treated with protein A (12 months). The lysis signals were calculated as described in section Data Evaluation. Increased lysis signals were explained by the use of a different complement source. (B) Binding of RBD-liposomes to immobilized 0 or 5 µg/mL ACE2, incubated for 3 h at RT and 300 rpm. Non-bound liposomes were washed away. Bound liposomes were lysed by a detergent and their fluorescence signal recorded. Enhanced binding after six months was due to varying ACE2 quality, decreased binding after one year was explained with slight degradation of the RBD at RBD-liposomes. Two-tailed, paired t-test for significance determination. Shown are mean ± SD, n = 3.

Patient Sample Complement Activity

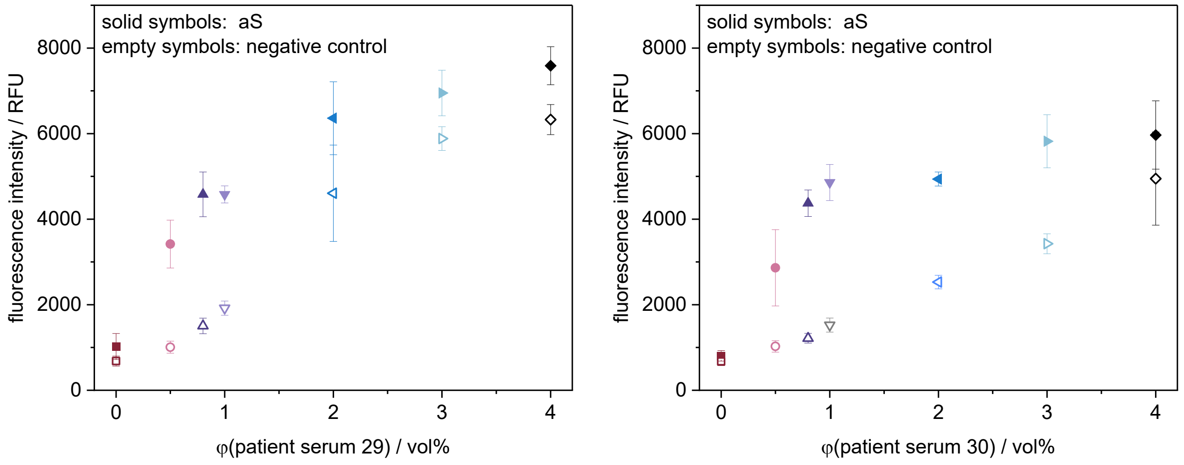

Figure S6: Two patient sera were tested in concentrations between 0 and 4 vol%. Samples were pre-incubated with RBD-liposomes for 20 min at 37 °C. Liposomes were then incubated for 60 min at 37 °C in the presence of 10 vol% canine complement source. Inactivated complement source was used as negative control. The endpoint raw data show that complement-active samples led to increasing background signals (negative control) with increasing sample concentration due to complement activation during the 20 min pre-incubation step and a previous inactivation of the patient complement system is required. Shown are mean ± SD, n = 3.

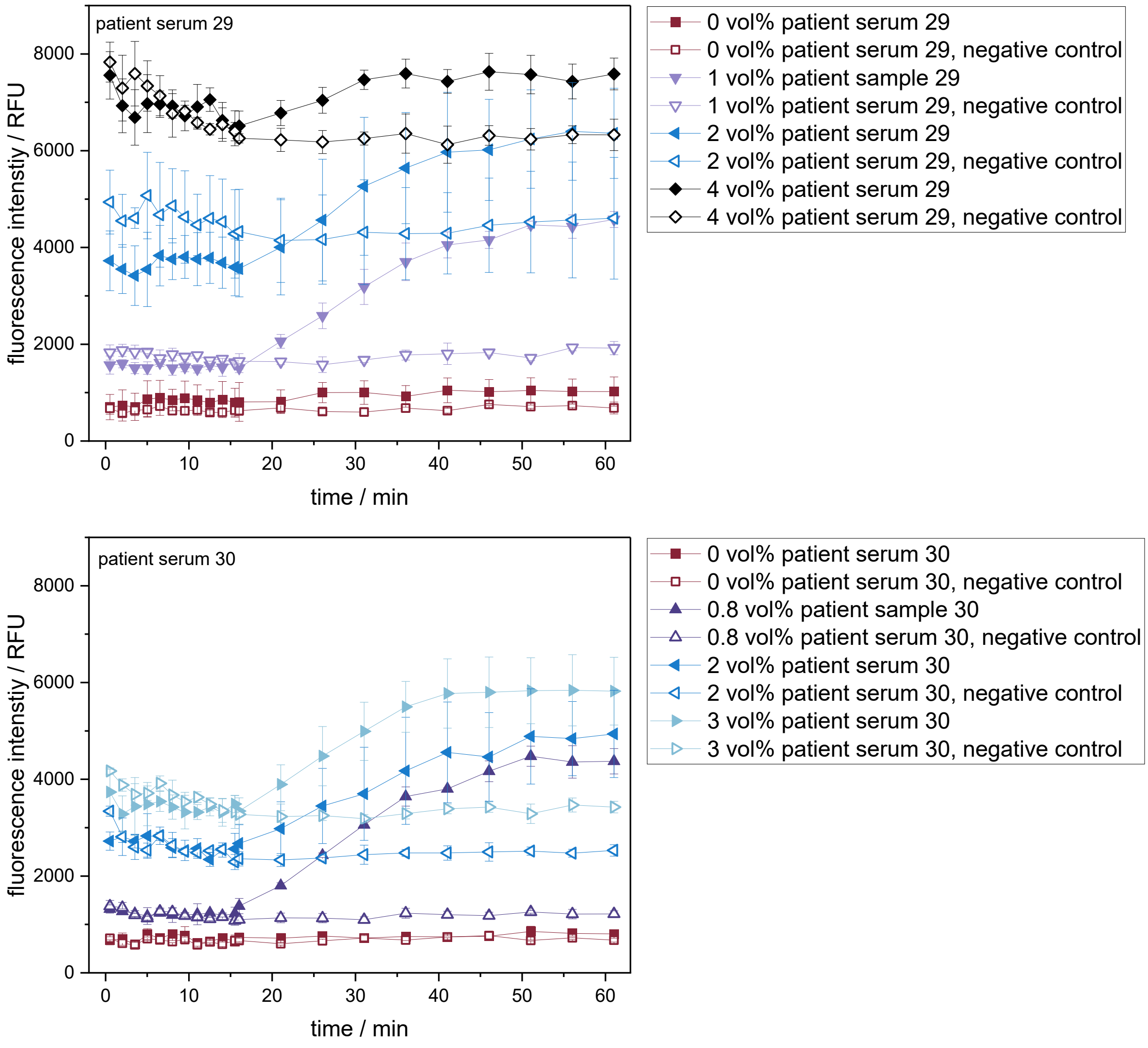

Figure S7: Two patient sera were tested in concentrations between 0 and 4 vol% (see Figure S6). Samples were pre-incubated with RBD-liposomes for 20 min at 37 °C. Liposomes were then incubated for 60 min at 37 °C in the presence of 10 vol% canine complement source. Inactivated complement source was used as negative control. The time-resolved raw data of four exemplary sample concentrations show that complement-active samples led to a shift in the background signals (negative control) with increasing sample concentration due to complement activation during the 20 min pre-incubation step and a previous inactivation of the patient complement system is required. Shown are mean ± SD, n = 3.

Heat-Inactivation of the Patient Sample

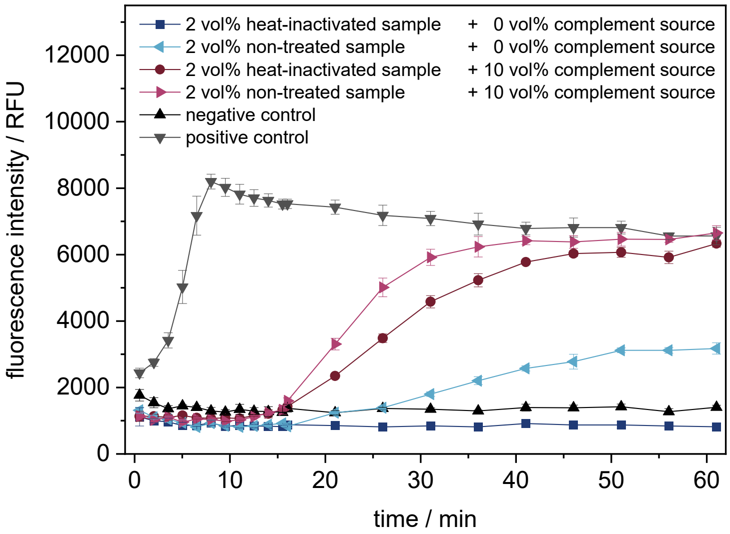

Figure S8: The inactivation of the complement system in the patient sample by treatment at 56 °C for 30 min was investigated. The patient sample was either heat-inactivated or not and then 2 vol% patient serum pre-incubated with RBD-liposomes for 20 min at 37 °C. Liposomes were then incubated for 60 min at 37 °C in the presence of 10 vol% human complement source with 4.5 µg/mL RBD for clearance of antibodies in the complement source (see Figure 4, A). Inactivated complement source was used as negative control, liposomes fully lysed by a detergent were used as positive control. The time-resolved raw data show that the non-treated sample led to complement-induced liposomes lysis in absence of human complement source, in contrast to the non-treated patient sample. In presence of the complement source, this results in different lysis signals. Thus, heat-inactivation of the patient samples is performed to exclude varying overall complement activity and to obtain the same conditions for all samples. Shown are mean ± SD, n = 3.

Canine Complement Source as Potential Alternative for Pandemic Human Source

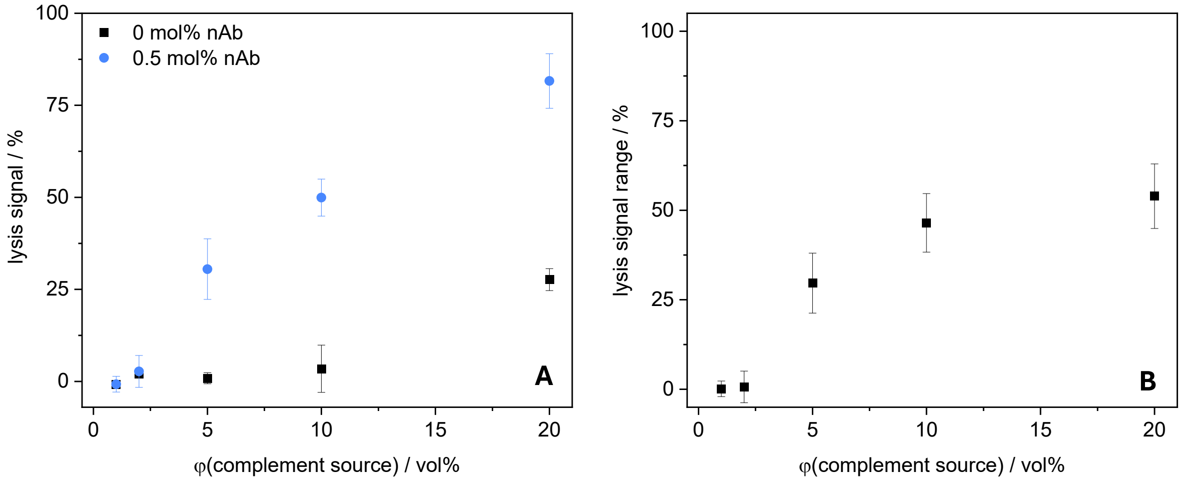

Figure S9: The use of canine complement source was investigated as an alternative for human complement source which contains anti-SARS-CoV-2 antibodies and the concentration optimized for maximum complement-induced lysis while maintaining liposome stealthiness. (A) Lysis signals of samples with and without 0.5 mol% neutralizing antibody (nAb) PA5-114451 for 1 h at RT and 300 rpm and various concentrations of canine complement source. (B) Lysis signal range of different complement concentrations as difference of the lysis signal of the sample incubated with 0.5 mol% antibody and the sample without antibody. Liposomes were then incubated for 60 min at 37 °C in the presence of 1–20 vol% of canine complement source. The lysis signals were calculated as described in section Data Evaluation. Shown are mean ± SD, n = 3.

Inactivation and Reactivation of Human Complement Source

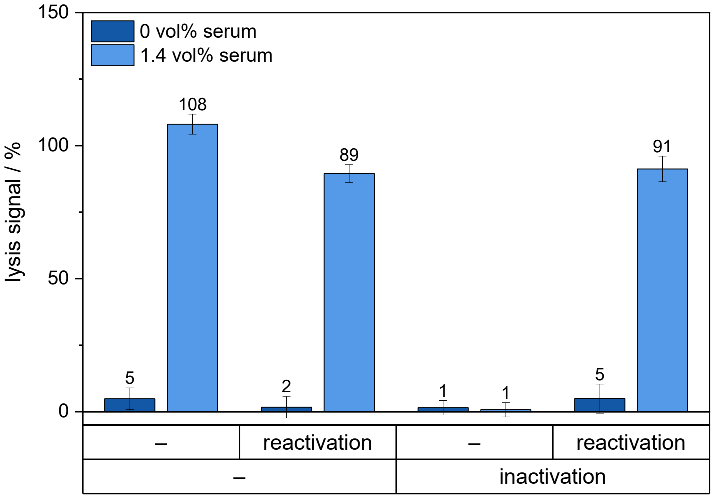

Figure S10: The temporary inactivation of the complement system in the human complement source was investigated to prevent non-specific complement activation. Human complement source was either non-treated or inactivated by addition of 1.6 mM EDTA per 10 vol% complement source and in a second step either reactivated by addition of 1.1 mM Ca^{2+} and 0.5 mM Mg^{2+} or not. RBD-liposomes were pre-incubated with or without 1.4 vol% serum for 20 min at 37 °C. Liposomes were then incubated for 60 min at 37 °C in the presence of 10 vol% human complement source. The lysis signals were calculated as described in section Data Evaluation. Shown are mean ± SD, n = 3.

Assay Range Depending on the Concentration of Complement Source

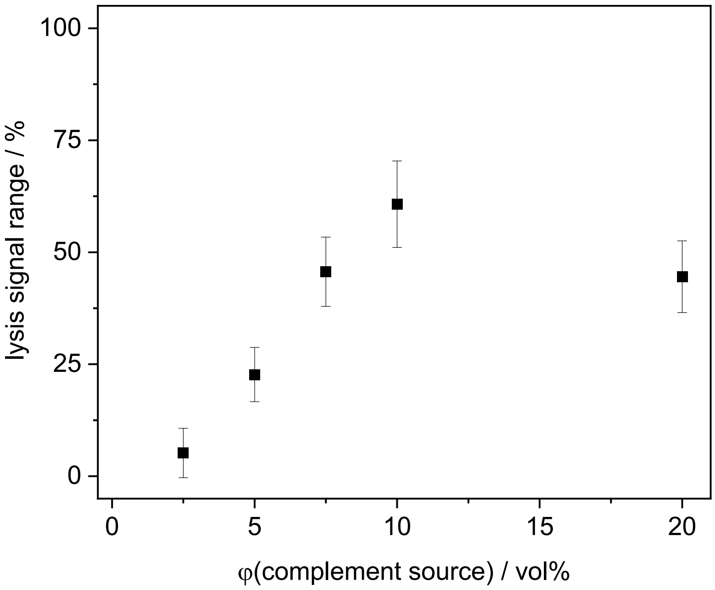

Figure S11: The concentration of human complement source treated with protein A was optimized for a maximum range in the lysis signal (lysis signal without sample subtracted from lysis signal with 1 vol% sample). At a concentration of 10 vol%, maximum complement induced lysis was observed while the liposome stealthiness was maintained (see Figure 3, C). RBD-liposomes were pre-incubated with serum for 20 min at 37 °C. Liposomes were then incubated for 60 min at 37 °C in the presence of 2.5–20 vol% of complement source treated with protein A. n = 3. The lysis signals were calculated as described in section Data Evaluation. Shown are mean ± SD, n = 3.

Seronegative Samples

Table S1: Background information of the SARS-CoV-2 negative sera 1-20 from the TiCoKo19 study including sex, age, BMI and bodyweight classification, previous diseases (autoimmune diseases, cancer, cardiovascular diseases), smoking habits and ELISA EC_{50} against Influenza A (A/England/195/2009 H1). No background information was available for samples 21-37.

ID	Sex	Age	BMI	Bodyweight Category	Auto-immune	Cancer	Cardio-vascular	Smoking	H1 EC_{50}
**1**	m	23	23.4	normal	-	-	-	current	251
**2**	f	16	27.5	overweight	-	-	-	never	374.4
**3**	f	82	30.4	adipositas	-	-	+	NA	1330
**4**	f	64	29.7	overweight	+	+	-	ex	961.4
**5**	m	52	31.5	adipositas	-	+	-	ex	497.5
**6**	m	52	26.5	overweight	-	-	-	never	694.7
**7**	f	66	22.8	normal	-	-	-	ex	358.5
**8**	m	81	24.9	normal	-	-	-	never	2053
**9**	m	22	26.6	overweight	-	-	-	never	2127
**10**	m	71	25.5	overweight	-	-	-	never	2774
**11**	m	51	27.8	overweight	-	-	+	never	195.9
**12**	m	73	26.6	overweight	-	-	+	ex	4969
**13**	f	17	20.2	normal	-	-	-	NA	398.5
**14**	m	54	30.3	adipositas	-	-	-	current	1157
**15**	f	18	19.4	normal	-	-	-	never	994.4
**16**	f	45	24.6	normal	+	-	-	current	196
**17**	f	56	25.1	overweight	-	-	-	current	66.78
**18**	m	20	21.9	normal	-	-	-	never	1495
**19**	m	61	25.3	overweight	-	-	+	ex	259.2
**20**	f	27	28.2	overweight	-	-	-	NA	630.8
**Sum/ Median**	**11 m 9 f**	**52**	**26.0**	**3/10/7**	**2**	**2**	**4**	**4/5/8**	**662.8**

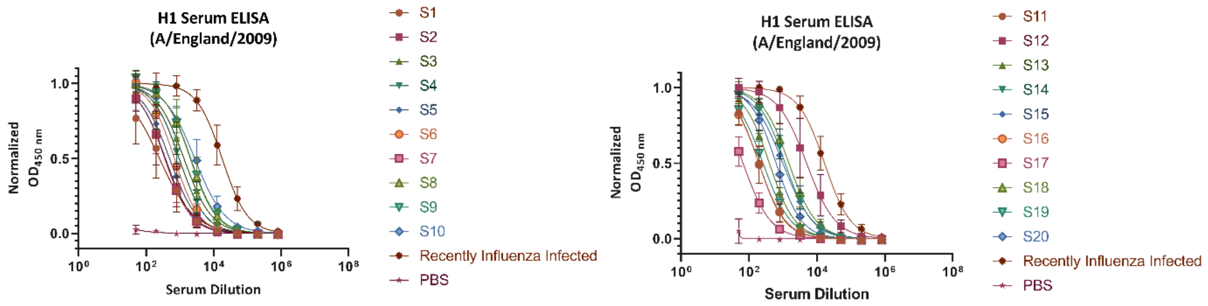

Figure S12: 20 anti-SARS-CoV-2 negative patient sera were screened for anti-Influenza (A/England/195/2009 H1) antibodies. 350 ng/well Influenza HA protein (A/England/195/2009) in PBS were coated on Nunc Maxisorp plates overnight, blocked for 1 h with 5 % skim milk powder, primary stained with a 4-fold serum dilution starting at 1:50 and secondary stained with polyclonal Rabbit anti-Human IgG-HRP (Agilent Technologies, P0214) in a 1:5000 dilution. OD at 450 nm was determined after 2-minute TMB incubation and stopping with 1 M H_2SO_4. Titration curves for each serum were determined in three independent measurements and normalized to mean PBS signal and the highest signal of the positive control serum (recently Influenza infected) on each plate.

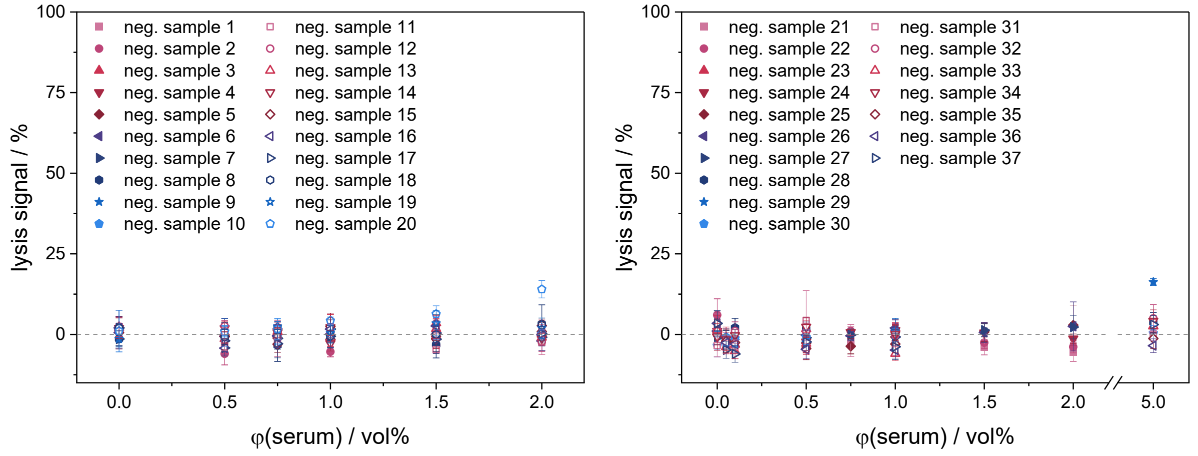

Figure S13: 37 seronegative patient sera were screened for anti-SARS-CoV-2 antibodies. The patient samples were heat-inactivated and then pre-incubated with RBD-liposomes for 20 min at 37 °C in various concentrations (0.5, 1, 5, 7.5, 10, 15, 20, 50 vol%). Liposomes were then incubated for 60 min at 37 °C in the presence of 10 vol% human complement source. The dashed line represents 0 % lysis signal. The lysis signals were calculated as described in section Data Evaluation. Two samples (no. 20, 29) gave false-positive signals. Shown are mean ± SD, n = 3.

Seropositive Samples

Table S2: Seropositive samples with known background (variant of infection or vaccination (A = Alpha, D = Delta, O = Omicron, X = no infection), times of vaccination) were screened in the liposome-based assay, ELISA and pVNT. 2 samples (no. 29 and 30) revealed the necessity of heat-inactivation and not used in the screening, see Figure S6/ Figure S7. Alpha-RBD was used in all assays. ID_{50} values of 2561 denote assay saturation at the lowest tested dilution of 2560.

**Sample ID**	**Variant**<br>**A = Alpha**<br>**D = Delta**<br>**O = Omicron**<br>**X = no infection**	**Times of Vaccination**	**EC_{50} / vol% (liposome-based assay)**	**ED_{50} / (ELISA, Alpha-RBD)**	**ID_{50} (pVNT, Alpha-RBD)**
**1**	A	1x	0.067 ± 0.0017	13930.3	2561
**2**	A	1x	0.073 ± 0.005	10763.7	2561
**3**	A	1x	0.060 ± 0.0013	11920.7	2561
**4**	A	1x	0.060 ± 0.003	6418.7	2561
**5**	A	2x	0.40 ± 0.06	683.8	1647
**6**	A	2x	0.3 ± 0.2	1615.3	1979
**7**	A	2x	0.18 ± 0.03	1432.3	2561
**8**	D	0x	>5<br>no exact determination possible	96.5	57.09
**9**	D	2x	2.1 ± 0.13	391.6	103.3
**10**	D	2x	0.62 ± 0.06	1053.3	116.2
**11**	D	2x	0.72 ± 0.08	522.8	113
**12**	D	2x	<0.5<br>no exact determination possible	5034.3	1309
**13**	D	2x	>5	106.5	35.64

			no exact determination possible		
**14**	D	2x	0.72 ± 0.06	454.3	150.1
**15**	D	2x	0.3 ± 0.15	1581	1040
**16**	D	0x	7.5 ± 0.7	149.4	86.54
**17**	D	2x	0.22 ± 0.03	4561.7	1951
**18**	D	1x	1.12 ± 0.09	532.3	125.9
**19**	D	1x	0.48 ± 0.04	1250.3	597.2
**20**	D	2x	0.080 ± 0.007	2300.3	1862
**21**	D	2x	0.08 ± 0.012	3632.3	2561
**22**	D	2x	0.14 ± 0.03	1502	1352
**23**	D	2x	0.33 ± 0.07	2370	1525
**24**	D	2x	0.31 ± 0.03	1231.7	1534
**25**	D	2x	4.1 ± 0.14	241	109.8
**26**	D	2x	0.3 ± 0.10	1237.2	1535
**27**	D	2x	0.36 ± 0.05	3040.3	1291
**28**	D	2x	0.24 ± 0.05	3711.3	1092
**29**	X	2x	No exact determination possible as sample was not heat-inactivated	Not determined	467.1
**30**	O	4x	No determination possible as sample was not heat-inactivated	Not determined	1741

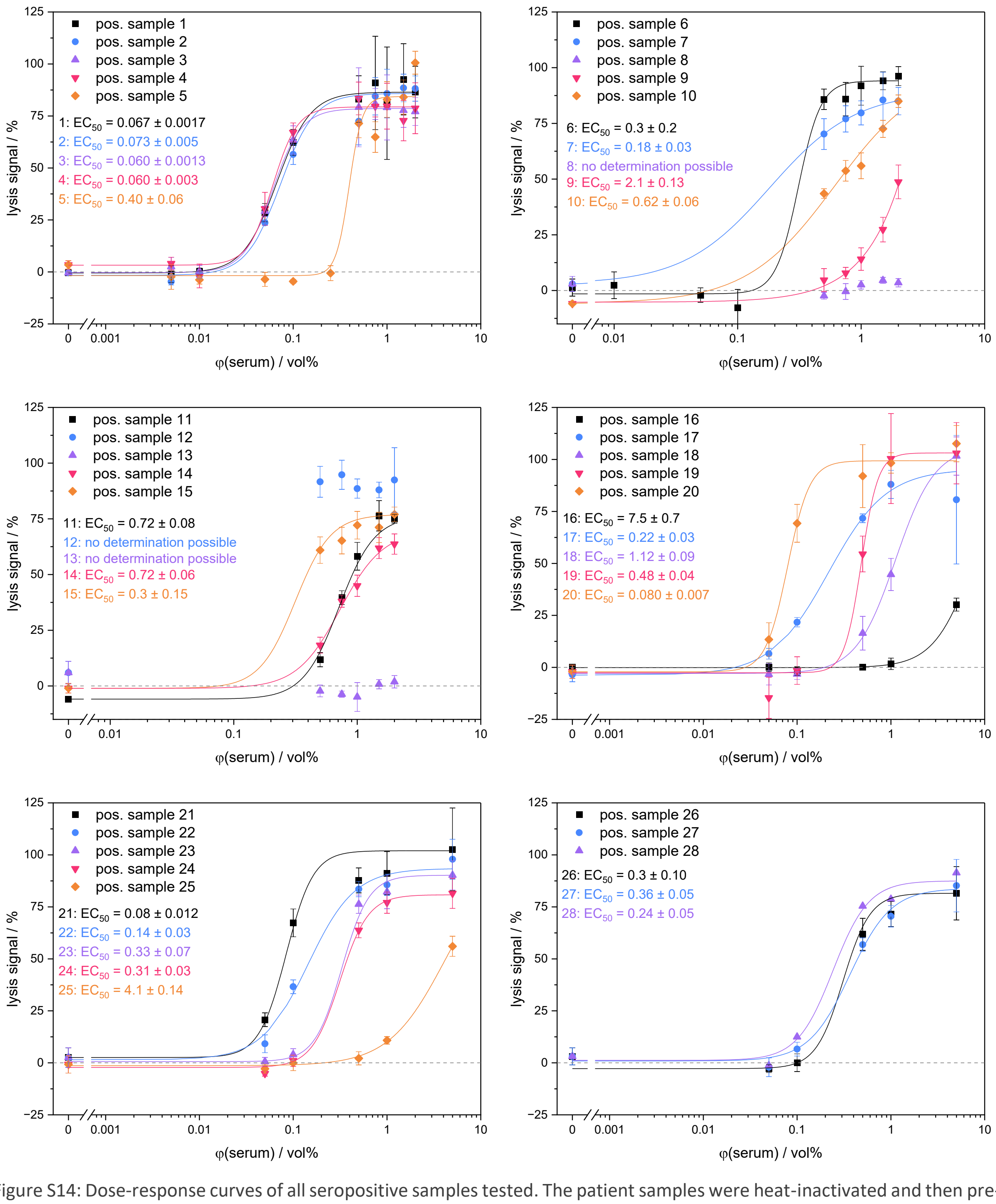

Figure S14: Dose-response curves of all seropositive samples tested. The patient samples were heat-inactivated and then pre-incubated with RBD-liposomes for 20 min at 37 °C in various concentrations (0.05–50 vol%). Liposomes were then incubated for 60 min at 37 °C in the presence of 10 vol% human complement source. The dashed line represents 0 % lysis signal. The lysis signals were calculated as described in section Data Evaluation. EC_{50} values and 95 % CIs were determined using a 4-parameter logistic regression curve fit as described in section Data Evaluation. Shown are mean ± SD, n = 3.

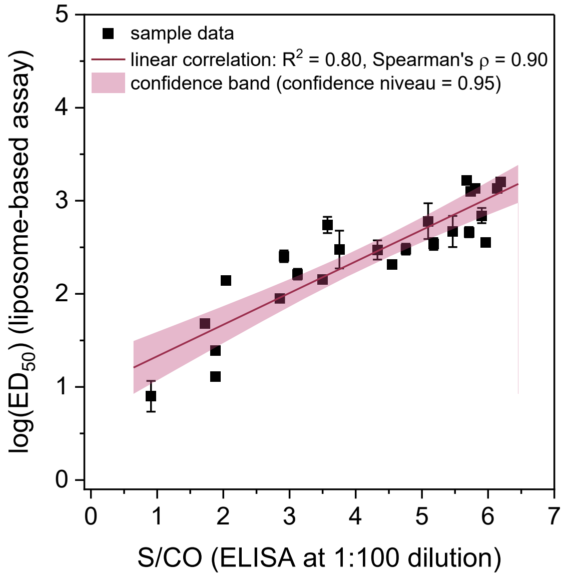

Figure S15: 28 seropositive human serum samples were screened and the results correlated to other assay formats: Correlation of $log_{10}(ED_{50})$ values from the liposome-based assay with the signal to cutoff ratios (S/CO) at a 1:100 dilution from the diagnostic ELISA.

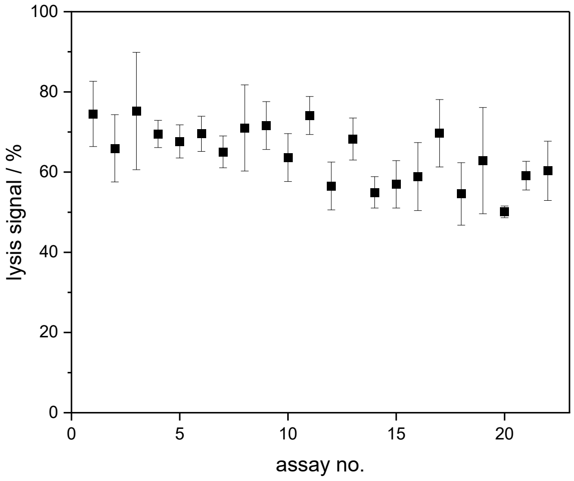

Figure S16: The reproducibility of the assay was investigated for 1.4 vol% of a positive control serum IRS45270 in 22 assays. The control sample was heat-inactivated and then pre-incubated with RBD-liposomes for 20 min at 37 °C (14 vol%). Liposomes were then incubated for 60 min at 37 °C in the presence of 10 vol% human complement source. The lysis signals were calculated as described in section Data Evaluation. n = 3.

Receiver Operating Characteristic (ROC) Curve

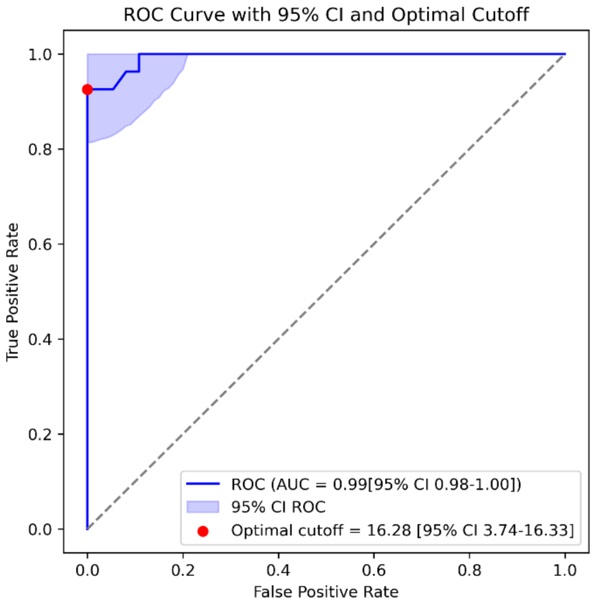

Figure S17: Receiver operating characteristic (ROC) analysis was performed to evaluate the binary discriminative ability of the liposome-based assay against the binary outcome as determined by the diagnostic ELISA. For dichotomous classification, the maximum mean liposome lysis signal for any sample concentration was used. Area under the ROC Curve (AUC) was calculated as a measure of the overall classification performance and the optimal cutoff was determined by Youden's J (sensitivity+specificity-1) on a fine threshold grid (1000 thresholds over score range), selecting the cutoff to maximize the statistic. Confidence intervals and uncertainty for the ROC, AUC, optimal cutoff and connected sensitivity and specificity were estimated by 2000 bootstrap resamples.
